# Recovery of valuable metals from spent lithium-ion batteries using microbial agents for bioleaching: a review

**DOI:** 10.3389/fmicb.2023.1197081

**Published:** 2023-05-31

**Authors:** Basanta Kumar Biswal, Rajasekhar Balasubramanian

**Affiliations:** Department of Civil and Environmental Engineering, National University of Singapore, Singapore, Singapore

**Keywords:** spent Li-ion batteries, cathode material, bioleaching, biohydrometallurgy, metal recovery, lithium and cobalt, sustainability, circular economy

## Abstract

Spent lithium-ion batteries (LIBs) are increasingly generated due to their widespread use for various energy-related applications. Spent LIBs contain several valuable metals including cobalt (Co) and lithium (Li) whose supply cannot be sustained in the long-term in view of their increased demand. To avoid environmental pollution and recover valuable metals, recycling of spent LIBs is widely explored using different methods. Bioleaching (biohydrometallurgy), an environmentally benign process, is receiving increased attention in recent years since it utilizes suitable microorganisms for selective leaching of Co and Li from spent LIBs and is cost-effective. A comprehensive and critical analysis of recent studies on the performance of various microbial agents for the extraction of Co and Li from the solid matrix of spent LIBs would help for development of novel and practical strategies for effective extraction of precious metals from spent LIBs. Specifically, this review focuses on the current advancements in the application of microbial agents namely bacteria (e.g., *Acidithiobacillus ferrooxidans* and *Acidithiobacillus thiooxidans*) and fungi (e.g., *Aspergillus niger*) for the recovery of Co and Li from spent LIBs. Both bacterial and fungal leaching are effective for metal dissolution from spent LIBs. Among the two valuable metals, the dissolution rate of Li is higher than Co. The key metabolites which drive the bacterial leaching include sulfuric acid, while citric acid, gluconic acid and oxalic acid are the dominant metabolites in fungal leaching. The bioleaching performance depends on both biotic (microbial agents) and abiotic factors (pH, pulp density, dissolved oxygen level and temperature). The major biochemical mechanisms which contribute to metal dissolution include acidolysis, redoxolysis and complexolysis. In most cases, the shrinking core model is suitable to describe the bioleaching kinetics. Biological-based methods (e.g., bioprecipitation) can be applied for metal recovery from the bioleaching solution. There are several potential operational challenges and knowledge gaps which should be addressed in future studies to scale-up the bioleaching process. Overall, this review is of importance from the perspective of development of highly efficient and sustainable bioleaching processes for optimum resource recovery of Co and Li from spent LIBs, and conservation of natural resources to achieve circular economy.

## 1. Introduction

Lithium-ion batteries (LIBs) are widely used in electric vehicles, energy storage systems, mobile phones, and other portable electronic devices for energy storage applications (Martins et al., 2021; Miao et al., 2022). The use of LIBs as energy storage devices is mainly due to their high energy density, high reliability, higher output voltage, fast charging ability, higher resistance to self-discharge, light weight and longer lifetime (Miao et al., 2022; Alipanah et al., 2023). There is a huge demand for LIBs (USD$36.7 billion in 2019), and it is projected to increase by nearly fourfold (i.e., USD$129.3 billion) by 2027 (Dyatkin and Meng, 2020). The global LIB production capacity is estimated to increase from 455 GWh in 2020 to 1,447 GWh in 2025, i.e., with a compound annual growth rate (CAGR) of 26% (Alipanah et al., 2021). Notably, China was the major producer of LIBs, e.g., in 2020, contributing to 77% of the total LIBs production globally (Alipanah et al., 2021). Due to explosive production and usage of LIB-based portable and non-portable devices, a huge amount of spent LIBs is generated (Golmohammadzadeh et al., 2022). The amount of spent LIBs generated has been estimated to reach 640,000 metric tons in China by 2025 (Yang et al., 2022), while in Australia, it is expected to reach 137,000 metric tons by the end of 2036 (Golmohammadzadeh et al., 2022). With the assumption that the average lifespan of LIBs used in automotive applications (e.g., electric vehicles) is 10 years, it is projected that 700,000 metric tons of LIBs will reach their end of life by 2025 globally (Alipanah et al., 2021).

The different components of spent LIBs and their corresponding percentage in the total weight are: cathode (35%), battery case (25–30%), anode (15–18%), electrolyte (11–12%), plastic materials (5–6%) and others (mass loss during treatment, e.g., drying, 3–4%) (Horeh et al., 2016; Heydarian et al., 2018). LiCoO_2_ (lithium cobalt oxide) is one of the most preferred cathodes than other lithium oxides-based cathodes and extensively used in portable electronic devices at the current time. The use of LiCoO_2_ is likely to continue in the future primarily due to its high energy density and longevity (Zeng et al., 2014; Zhang et al., 2022). The two major metals in spent LIBs (with LiCoO_2_ as cathode material) are cobalt (Co) which is detected up to 30.4% and lithium (Li) which is found up to 10.3% of the total weight of spent LIBs (Heydarian et al., 2018). Other elements detected in spent LIBs at varying concentrations include nickel (Ni), manganese (Mn), copper (Cu), aluminum (Al) and iron (Fe) (Table 1). The concentration of Cu, Ni and Mn in spent LIBs varies between 6–12%, 5–10% and 5–11%, respectively (Roy et al., 2021a; Ratnam et al., 2022). The concentration of these elements varies in natural ores depending on the types of minerals (e.g., primary vs. secondary minerals, or based on the chemical groups, e.g., sulfide, arsenide, carbonate, oxide-containing ores) (Dehaine et al., 2021). For example, carrollite which is a sulfide-containing mineral contains 28.56% Co, 20.53% Cu and 9.48% Ni, while skutterudite (arsenide containing mineral) contains only 17.95% Co and 5.96% Ni (Dehaine et al., 2021). There are variations of metal contents in spent LIBs which is possibly due to variations of manufactory (battery chemistry) (Xin et al., 2016; Sethurajan and Gaydardzhiev, 2021). As a result of a significant increase of LIBs production worldwide, the price of some of the metals used in LIBs considerably increased, e.g., the Co price increased nearly 4 times in the last 2 years, from US$ 22 /kg to US$ 81 /kg (Du et al., 2022). According to a recent study, the price of Li also increased by three-times (Ratnam et al., 2022). Fan et al. (2020) reported that the average price of Co was US$ 75,991.27/ton in 2018, which was 10 and 5 times higher than that of Mn and Ni, respectively. With the current trend of increasing production of LIBs, nearly 70% of the global Co reserves is expected to be spent for battery production by 2040, and the demand for LIBs is projected to go beyond its supply by 2030 (Alipanah et al., 2023). At present, 35% of the globally produced Li and 25% of globally produced Co are utilized for LIBs production and it is estimated that the Li consumption could be doubled (66%) by 2025 (Swain, 2017; Golmohammadzadeh et al., 2022). It should be noted that the global reserves for Co and Li are limited to nearly 145 million tons and 62 million tons, respectively (Fan et al., 2020).

**TABLE 1:** Metal contents in spent Li-ion batteries (LIBs) reported in various studies.

	Quantity of key metals in spent LIBs (%, w/w)							
**Method**	**Co**	**Li**	**Cu**	**Mn**	**Fe**	**Al**	**Ni**	**References**
Chemical digestion	22.05	3.03	2.55	0.44	0.38	4.27	0.05	Roy et al., 2021b
Chemical digestion	16.0	2.1	0.62	0.07	–	–	0.04	Biswal et al., 2018
Chemical digestion	7.44	5.16	2.82	9.1	0.97	0.62	15.96	Jegan-Roy et al., 2021
Chemical digestion	16.54	2.22	5.93	21.31	0.04	9.12	2.56	Horeh et al., 2016
Chemical digestion	30.4	10.3	0.6	5.2	2.2	0.3	8.2	Heydarian et al., 2018
Chemical digestion	15.6	4.2	8.1	20.5	0.5	4.8	15	Xin et al., 2016
Chemical digestion	–	4.5	5.4	26.5	2.3	5.2	–	Xin et al., 2016
Chemical digestion	–	5.0	7.6	–	25	4.4	–	Xin et al., 2016
Chemical digestion	–	2.76	–	–	–	–	–	Badawy et al., 2013
Chemical digestion	31.18	7.04	0.22	5.02	0.93	0.61	9.07	Ghassa et al., 2021
Chemical digestion	46	2.0	–	0.85	–	1.5	2.7	Alavi et al., 2021
Chemical digestion	20.46	3.74	–	23.65	–	6.14	3.45	Nazerian et al., 2023
XRF	17.11	–	6.6	22	0.19	9.45	2.82	Horeh et al., 2016
XRF	48	–	–	0.87	–	1.52	2.8	Alavi et al., 2021
XRF	53.82	–	0.20	1.67	–	0.12	2.99	Hariyadi et al., 2022b
EDX	48.5	3.37	–	23.9	0.14	–	24.1	Li et al., 2013
EDX	48.5	3.37	–	23.9	0.14	–	–	Zeng et al., 2012
EDX	38.54	–	–	1.03	–	2.08	–	Hariyadi et al., 2022a

In chemical digestion method, metal analysis was done mainly using the inductively coupled plasma optical emission spectroscopy (ICP-OES) instrument. XRF, X-ray fluorescence spectroscopy; EDX, energy-dispersive X-ray spectroscopy analysis.

Disposal of LIBs used in electronic applications as part of various solid waste streams due to their limited life span (e.g., the typical life span of LiCoO_2_-based LIB is 1–2 years, 500–1,000 cycles) is an issue of concern (Aboelazm et al., 2021; Lin et al., 2021; Yu et al., 2022). The reason for this concern is that improper management and disposal of untreated spent LIBs could pose negative effects on human health, environment and ecosystems (Huang et al., 2019). Metals namely Co and Ni present in LIBs are categorized as carcinogenic and mutagenic materials. Furthermore, the toxic organic electrolytes/solvents used in LIBs could have adverse impacts on the human health and environment (Fan et al., 2020). The polymers like polyethylene and polypropylene used in separators could also pose negative environmental effects (e.g., cause microplastic pollution) (Golmohammadzadeh et al., 2022; Ratnam et al., 2022). The spent LIBs are considered as secondary source of metals, and sometimes, the metal quantity (e.g., concentration) is higher than what is available in the concentrated ores or natural ores (Xi et al., 2015). In view of the limited supply of Li and Co, it is important to reduce the high demand on the natural metal resources, save the valuable metals present in the spent LIBs and mitigate environmental pollution caused by the hazardous components of spent LIBs, spent LIBs should be appropriately handled and recycled.

Recycling could play a major role in the overall sustainability of future LIBs by recycling the secondary metallic resources and also contribute to the circular economy (Golmohammadzadeh et al., 2022). It is reported that recycling and reuse of valuable metals namely Co and Ni from spent LIBs could save 51.3% of natural resources and reduce the mining of metals from the virgin mineral sources (Dewulf et al., 2010). The recovered valuable metals from spent LIBs can be reused in LIBs or other products including supercapacitors (Ratnam et al., 2022). The commonly used recycling methods for spent LIBs include direct recycling, pyrometallurgy, hydrometallurgy and biohydrometallurgy (bioleaching) (Golmohammadzadeh et al., 2022; Roy et al., 2022). The major advantages and disadvantages of various types of recycling methods are given in Table 2. Among the various types of recycling methods, bleaching is cost-effective, environmentally friendly, simple in operation and less energy intensive (Golmohammadzadeh et al., 2022; Mokarian et al., 2022). As of 2018, the recycling rate of LIBs was only 8.86% (Mao et al., 2022), and the global rate of Li recycling is even lower (i.e., < 1%) (Swain, 2017).

**TABLE 2:** Comparison of potential advantages and disadvantages of commonly used LIB recycling methods (Roy et al., 2021a, 2022; Golmohammadzadeh et al., 2022; Mao et al., 2022).

Recycling method	Advantages	Disadvantages
Direct recycling	Practically feasible to recover different components of spent LIBs	Recovered materials may not perform like virgin material
	Active materials can be recovered with original chemical structures	Mixing of cathode material could decrease the quality of the recycled material
	Energy efficient	Regeneration process is not developed yet
	Economically feasible	It is remains at laboratory-scale, not applied industrial-scale
Pyrometallurgy	All types of spent LIBs with different chemistries can be recycled without sorting or treatment	Loss of lithium in the recycling process (formation of slag)
	Recovery of metals as alloys due to direct melting	Not suitable to recover Fe, Al and Mn
	Fast and high efficiency (high recovery of various metals including Co, Co and Ni)	High energy demand (operation and maintenance)
	Direct feeding into furnace allows practical large-scale operation	Emission of dust and toxic gases (e.g., CO_2_)
	No wastewater generation	Purity of final product is expected to be low
	Lower possessing steps compared to the hydrometallurgical method	Not a flexible process
Hydrometallurgy	Applicable to any LIBs chemistries	Recycling involves complex and multi-steps operation
	Process is flexible to recover a specific target metal	Need pre-treatment of spent LIBs (e.g., discharging, shredding, sieving and separation)
	High process efficiency with high purity of the extracted metals	Changes of cathode material structure due to acid treatment
	High environmental sustainability due to less emission of hazardous gases	Formation of large volume of corrosive (acidic) wastewater
	Cost-effective and less energy demand	Need additional capital investment for treatment of wastewater
Biohydrometallurgy (Bioleaching)	Environmentally friendly	Slow process kinetics
	Low operation cost and energy requirement	Long processing time
	Minimal use of chemical reagents	Not feasible in high toxic environment
	Achieve high efficiency at low metal concentration	Low efficiency at higher pulp density
	Less issue of toxic gas generation	Hard process control measures

Although LIBs dominate in the various energy markets (e.g., from portable electronic devices to electric vehicles) (Wu Y. et al., 2019), from the sustainability perspectives (e.g., to reduce carbon footprint), recently increasing interest is given on the development of renewable/green energy (e.g., solar, wind and biomass-based energy) (Qazi et al., 2019; Ray et al., 2022). Specifically, in the biomass-based energy, diverse microalgae species are explored for their potential as a feedstock for biofuel production (Nayak et al., 2018, 2020). For the sustainable energy systems, biomass-based electrode materials (e.g., anode: bio-graphite) and bio-based solid electrolytes are explored in LIBs (Sagues et al., 2020; Raj et al., 2022). According to Sagues et al. (2020) the softwood-derived bio-graphite in a LIB cell shows 89% capacity retention over 100 cycles and more than 99% coulombic efficiency. Another study reported that the use of carbonated soybean oil-based electrolyte in LFP batteries exhibited the gravimetric capacity of 112 and 157 mAh/g at room temperature and 60 °C, respectively (Raj et al., 2022).

To understand the current state of knowledge on the recycling of spent LIBs, this review comprehensively analyzed the publication trend in the last 10 years (2013–2022) using the scientific database (e.g., Scopus) (Supplementary Figure 1). The two keywords used in the Scopus search engine are “spent Lithium-ion batteries” and “recycling” which resulted in 1,268 publications with only 15 publications in 2013, but 360 publications in 2022 (i.e., increased by 24 times). The continuous increase of publications pertaining to spent LIBs in the past 10 years indicates that there is an increasing interest among scientific communities to develop novel and sustainable technologies for the recycling of spent LIBs to recover valuable metals present in spent LIBs and contribute to environmental protection. A major fraction of these publications is related to the recycling of spent LIBs using the pyrometallurgy, hydrometallurgy or direct recycling. Previous studies have also reported that the above three methods are widely used for the recycling of spent LIBs (Zeng et al., 2014; Mao et al., 2022). In terms of the distribution of publications in various countries, around 60% of articles were published by researchers in China, followed by the United States (9%) and India (6%). Further analysis in the Scopus database employing the keywords namely “spent Lithium-ion batteries,” “bioleaching” and/or “biohydrometallurgy” revealed a total of 53 publications (37 journal articles, 8 review articles, 6 book chapters and 2 conference papers), indicating that bioleaching is getting much attention as an emerging environmentally friendly method. The flowchart for the review methodology is presented in Supplementary Figure 2. However, it should be noted that bioleaching is largely explored in the lab-scale mode for the recovery of valuable metals from spent LIBs. The most articles included in each subsection are mostly peer-reviewed articles published in 2013–2022 which are collected from the various online scientific database namely Scopus, Web of Science, and Google Scholar by using the keywords relevant to the particular section. Additionally, the relevance and data quality were further checked by reading the abstract and/or specific sections of the article.

Our literature review shows that a few review papers dealing with bioleaching of valuable metals in spent LIBs have been published (Moazzam et al., 2021; Roy et al., 2021a,^2022^; Sethurajan and Gaydardzhiev, 2021) among which one review mainly focused on the Li bioleaching (Moazzam et al., 2021). However, limited information is available on the comparative evaluation on the performance of bacterial and fungal-based bioleaching for recovery of major elements such as Co and Li from spent LIBs. Discussion on the quality and quantity of metabolites (bio-acids) produced due to interactions of LIB components (e.g., metals) with bacteria or fungi which drive the bioleaching process is scant. Application of possible biological and/or chemical methods for the recovery of Co and Li from the aqueous bioleaching media (e.g., transformation of dissolved metals into solid form through precipitation) was not sufficiently addressed in the past reviews. Understanding of the bioleaching kinetics is important to optimize the process performance which was found to be missing in the earlier reviews. From the circular economy perspective, critical discussion on the sustainability of the bleaching method for the recovery of valuable metals from spent LIB is necessary, but this was not considered previously.

The main objective of this review is to comprehensively analyze the recent developments in the literature on the application of microbial agents namely bacteria and fungi for the recovery of valuable metals (mainly Co and Li) from spent LIBs. The influence of various factors including bioleaching conditions (e.g., leaching medium pH, pulp density, aeration and substrate/energy source concentrations) and spent LIBs characteristics (e.g., powder particle size) on the recovery of valuable metals was assessed. Insights into microbe- metal interactions and the associated bioleaching mechanisms are presented. The sustainability of the bioleaching method is critically discussed. The key knowledge gaps that currently exist in literature and future research directions for further development of the bioleaching method with improved efficiency and sustainability are highlighted.

## 2. Overview of the development and chemistry of Li-ion batteries (LIBs)

The initial discovery of LIBs was done in 1970s. However, the first commercial LIB was produced by Sony in 1991 (Baum et al., 2022; Yang et al., 2022). In 2019, three scientists namely John B Goodenough, M Stanley Whittingham, and Akira Yoshino won the highly prestigious Nobel Prize in Chemistry for their pioneering works on the development of LIBs (Kamat, 2019; Service, 2019). In the battery technology, the key motivation is to use Li metal in the cathodic materials since Li is the most electropositive (–3.04 V) and lightest metal (molecular weight: 6.94 g/mol and specific gravity: 40.53 g/cm^3^), therefore enabling the design of storage systems with high energy density (Tarascon and Armand, 2001). The key chemical reactions involving in the primary non-rechargeable LIBs (e.g., the common Zn/MnO_2_ “Alkaline” cell) (Eqs. 1, 2) (Huggins, 2016; Roy et al., 2022) and secondary rechargeable LIBs (Eqs. 3, 4) are presented below (Moosakazemi et al., 2022; Roy et al., 2022). Additionally, the chemical reactions are involved in charge and discharge processes in LIBs having LiCoO_2_ as the cathode and graphite as the anode are presented in Eq. (5) (Zeng et al., 2014).

### 2.1. Primary non-rechargeable LIBs


(1) $$L\,i\to L\,i^{+}+e^{-}\,\left(A\,n\,o\,d\,e\right)$$

(2) $$L\,i^{+}+e^{-}+M\,n\,O_{2}\to L\,i\,M\,n\,O_{2}\,\left(C\,a\,t\,h\,o\,d\,e\right)$$

### 2.2. Secondary rechargeable LIBs


(3) $$L\,i\,C_{6}\,\left(L\,i\,t\,h\,i\,a\,t\,e\,d\,g\,r\,a\,p\,h\,i\,t\,e\right)↔C_{6}+L\,i^{+}+e^{-}$$

(4) $$L\,i\,C\,o\,O_{2}↔L\,i_{1-x}\,C\,o\,O_{2}+x\,L\,i^{+}+x\,e^{-}$$

(5) $$LiCoO_{2}+C_{6}\overset{Ch\arg e}{\underset{Disch\arg e}{↔}}Li_{\left(1-x\right)}CoO_{2}+Li_{x}C_{6}$$

**FIGURE 1:**
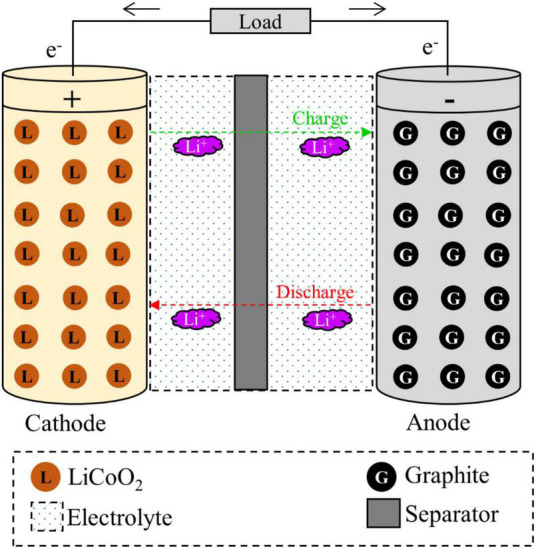
Schematic diagram for the different components of a typical LIB cell.

**FIGURE 2:**
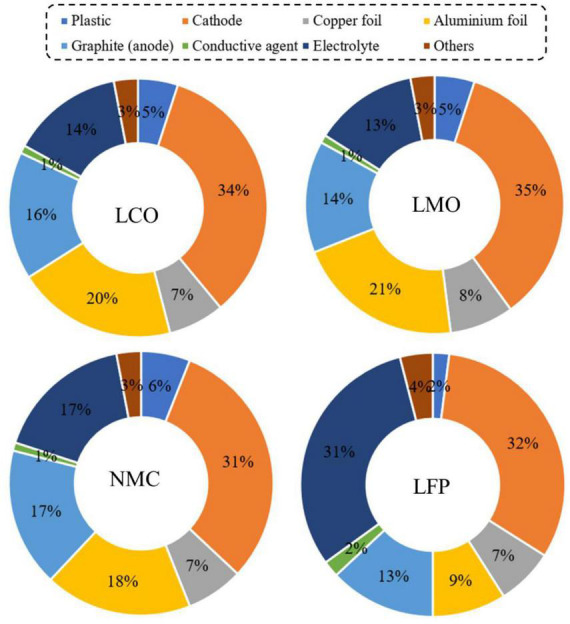
Weight fraction (wt %) of different components of LIBs with various cathode materials [adapted and modified from a previous study (Duan et al., 2022)]. LCO, LiCoO_2_; LMO, LiMn_2_O_4_; NMC, LiNi_*x*_Co_*y*_Mn_*z*_O_2_; and LFP, LiFePO_4_.

The key components of a typical LIB include anode (negative electrode–natural or synthetic graphite), cathode (positive electrode–different formulations of Li-based metal oxide), separator (electrolyte resistant polymers, e.g., polypropylene or polyethylene), electrolyte (lithium salts dissolved in an organic solvent e.g., LiPF_6_, LiBF_4_, etc.) and battery casing materials, and aluminum and copper foil (Golmohammadzadeh et al., 2022; Roy et al., 2022; Alipanah et al., 2023). The schematic diagram of a typical LIB cell with different components is presented in Figure 1. The weight fraction of various components of a LIB cell is given in Supplementary Table 1. A typical LiCoO_2_-based LIB contains 5% plastic, 34% LiCoO_2_ (cathode), 16% graphite (anode), 7% copper foil, 20% aluminum foil, 1% conductive agent, 14% electrolyte and 3% others (Figure 2) (Duan et al., 2022). The variation of the percentage of different components in LIBs could be due to LIBs production from different manufacturers (Zeng et al., 2014). Based on the shape, LIBs are categorized into four types including (1) cylindrical, (2) coin, (3) prismatic, and (4) thin and flat LIBs (Tarascon and Armand, 2001). Graphite is commonly used as the anode in LIB with the theoretical specific capacity of 372 mAh/g (Griffiths, 2016). On the basis of battery chemistry, different cathode materials are used in LIB namely LCO: lithium cobalt oxide (LiCoO_2_), NMC: lithium nickel manganese cobalt (LiCo_*x*_Mn_*y*_Ni_1–*x*–*y*_O_2_), LMO: lithium manganese oxide (LiMn_2_O_4_), LFP: lithium iron phosphate (LiFePO_4_), and NCA: lithium nickel cobalt aluminum oxide (LiNi_*x*_Co_*y*_Al_*z*_O_2_) (Alipanah et al., 2021; Baum et al., 2022). The characteristics of these cathodic materials are presented in Table 3. According to the life cycle assessment (LCA) of the LIB, production of 1 Wh storage capacity of LIB is linked to a cumulative energy demand of 328 Wh and emission of 110 gCO_2_eq of greenhouse gas (GHG) (Peters et al., 2017). The total cost for the production of one ton of LIB is US$ 77,708, and among the various components, the cost of the cathode material (e.g., LiCoO_2_, US$ 2,946) is higher than other parts (Gratz et al., 2014) (Supplementary Figure 3). Due to considerable progress on LIB research and developments, the price of LIB is gradually decreasing, e.g., from 3.17 $/Wh in 1991 to 0.28 $/Wh in 2005 (Vanitha and Balasubramanian, 2013). Among these cathodes, LiCoO_2_ is widely used in commercial applications (specifically in portable electronics) than others because LiCoO_2_ is relatively thermally more stable and has high energy density than other types of batteries (Tarascon and Armand, 2001; Baum et al., 2022). For the synthesis of LIBs, Li and Co are in greater demand compared to other metals due to their low relative abundance (Fan et al., 2020).

**TABLE 3:** Characteristics of cathode materials used in LIBs and their typical applications (Vanitha and Balasubramanian, 2013; Griffiths, 2016; Harper et al., 2019; Alipanah et al., 2021; Roy et al., 2022).

Cathode type	Chemical formula	Structure type	Energy density (Wh/kg)	Voltage (V)	Capacity (mAh/g)	Power density (W/kg)	Safety/stability	Calendar lifespan	Cycle lifespan	Year introduced	Key application
Lithium cobalt oxide (LCO)	LiCoO_2_	Layered	203	4.40	274	20.3	Low	Poor	500–1,000	1991	Portable electronics (e.g., mobile phone and laptop)
Lithium iron phosphate (LFP)	LiFePO_4_	Olivine	157.5	3.5	170	15.75	High	Good	2,000	1996	Electric vehicles, power tools, etc.
Lithium manganese oxide (LMO)	LiMn_2_O_4_	Spinel	144	4.1	148	14.4	Medium–high	Poor	300–700	1996	Electric vehicles, medical devices, e-bikes/scooters, power tools, etc.
Lithium nickel cobalt aluminum oxide (NCA)	LiNi_*x*_Co_*y*_Al_*z*_O_2_	Layered	200–260	3.60	200	–	Medium	Poor–good	500	1999	Electric vehicles, medical devices, laptops, etc.
Lithium nickel manganese cobalt (NMC)	LiCo_*x*_Mn_*y*_Ni_1–x–y_O_2_	Layered	155	4.6	200	25.5	Low–medium	Good	1,000–2,000	2008	Electric vehicles, power tools, e-bikes, etc.

## 3. Pre-processing/pre-treatment of spent LIBs

Lithium-ion batteries (LIBs) have complex chemistry and structural configurations. Hence, pretreatment is applied for disintegrating this complex structure so that the downstream resource recovery processes would be easier (Premathilake et al., 2023). The pre-treatment has several benefits including enhancement of metal recovery rate, decrease of energy consumption, reduction of environmental risks and avoidance of safety risks (Hua et al., 2020). The pre-treatment process consists of the following five stages namely (1) sorting, (2) stabilization/discharge, (3) dismantling/disassembly (4) grinding/crushing and (5) separation (Hua et al., 2020; Ali et al., 2022). The sorting of spent LIBs is carried out based on the physical appearance (shape, size, density and magnetic properties) and chemistry (type of cathode materials) (Ali et al., 2022). Batteries can be sorted based on electrical parameters (static and dynamic) namely internal resistance, voltage, self-discharge rate and discharge capacity (Ali et al., 2022). Automatic sorting methods such as X-ray sensors and optical sensors are also developed (Yu et al., 2021). Spent LIBs for recycling may contain a small amount of residual charges which may cause spark and explosion during the dismantling of batteries due to the reaction of Li with atmospheric oxygen (Premathilake et al., 2023). Discharge is a process to stabilize the spent LIBs by removing the residual energy to eliminate short circuit/explosion in the downstream processes of recycling (Hua et al., 2020; Du et al., 2022). The spent batteries are discharged using various methods to drain out the residual charge to less than 0.5 V to avoid the occurrence of any fire and explosion (Roy et al., 2022). The brine/electrolyte method (using NaCl or Na_2_SO_4_ salt solution) is commonly applied for stabilization of spent LIBs. Other methods used in discharging/stabilization include electrical/ohmic discharge (using an external circuit), and cryogenic discharge (e.g., using liquid nitrogen) and thermal deactivation (heating at 100–150°C) (Yu et al., 2021; Ali et al., 2022). Disassembling of spent LIBs is commonly done in two ways namely manual disassembly (mainly employed in laboratories) and automatic disassembly (e.g., large-scale industrial recycling) (Du et al., 2022). Manual dismantling (physical teardown) is done using mechanical tools such as screwdrivers, pliers, bolt cutters, knives and saws. However, it may cause several safety problems and environmental effects (Premathilake et al., 2023). In the disassembling process, different components of a spent LIB cell including outer metal casing, plastic materials, separator, cathode, anode and other materials (glue, binder, electrolytes, wire, etc.) are separated, and taken for further treatment wherever necessary (Du et al., 2022). Crushing is a size reduction process in which shredding, hammer milling and granulating can be applied based on the size and shape requirement of the next step of the separation process (Ali et al., 2022). Crushing can be done in two ways such as dry crushing (without addition of water) or wet crushing (presence of water or other solution which inactivate lithium) (Yu et al., 2021). Mechanical crushing is commonly used, and high efficiency can be achieved by using appropriate tools. The mechanical crushing enhances the contact surface of active cathode materials, and hence optimization of the recycling process is needed (Guimarães et al., 2022). A study evaluated the performance of three different types of grinding methods such as hammer, ceramic balls and knife mills for removal of electrode materials from spent LIBs (Takahashi et al., 2020). Notably, knife mill was effective for maximum recovery of cathode materials (i.e., achieved 11.2 wt % Co recovery) from the battery.

In the separation process, different parts of the crushed spent LIBs are separated based on their physicochemical properties such as size, density, hydrophobicity and ferromagnetism (Hua et al., 2020). The main aim of separation followed by purification is to separate active cathode materials (black mass) from other parts of spent batteries to achieve high recovery of valuable metals. The following separation processes are applied: (1) particle size fraction/sieving separation, (2) density/gravity separation (3) froth flotation separation, (4) magnetic separation and (5) electrostatic and eddy current separation (Ali et al., 2022). Other methods include mechanical separation (grinding and ultrasonic cleaning for separation of cathodes from foils), chemical dissolution (e.g., dissolution of organic binder by organic solvent and alkaline leaching) and thermal separation (high-temperature for separation of binder attached to the foils) (Yu et al., 2021). Based on the particle size distribution, the crushed material can be broadly divided into two fractions: fine fraction which mainly contains the active cathode materials, while the coarse fraction mostly consists of plastics, casing materials and separators (Premathilake et al., 2023). The mechanical separation is cost-effective and simple in operation, but results in low separation efficiency (Yu et al., 2021). However, organic solvent dissolution results in good separation and high recovery efficiency. The pre-processed/pre-treated spent LIB materials (in powder form) are taken for recycling of valuable metallic resources using various methods.

## 4. Overview of various methods for recycling of spent LIBs

The pre-treated spent LIB materials (mainly cathodic material) are taken to the next step of recycling process for the extraction of valuable metals. The following four methods such as direct recycling or three metallurgical-based methods (pyrometallurgy, hydrometallurgy and biohydrometallurgy) are commonly applied for the recovery of valuable metals from spent LIBs. In the direct recycling process, battery materials (e.g., cathode) are recovered with no or minimal change of their original chemical structure, and it is mainly carried out by physical and magnetic separation (Hua et al., 2020; Ali et al., 2022). Additionally, the surface and bulk properties of active battery materials can be restored using the chemical processes namely re-lithiation or hydrothermal methods (Ali et al., 2022). Pyrometallurgy (thermal processing) is a high temperature (500–1,000°C) thermal treatment which converts metal containing battery components into metallic alloy (Hua et al., 2020; Ali et al., 2022). Metals are converted into metal oxides. The pyrometallurgical process involves three main steps including pre-heating, plastic burnings and metal reducing. The thermal pre-treatments employed for the recovery of cathode materials include incineration, calcination and pyrolysis, and the enriched metals are processed using the roasting or smelting processes (Makuza et al., 2021). The efficiency of the pyrometallurgical method depends on various factors namely processing temperature, residence time, flux addition and types of purge gas (Makuza et al., 2021). Although pyrometallurgical process is industrially viable for large-scale recycling of spent LIBs, it shows poor performance toward Li recovery (Georgi-Maschler et al., 2012).

In hydrometallurgical method (aqueous processing), the valuable metals present in the cathodic materials are dissolved into a liquid at low temperature, followed by separation and purification to recover valuable metals (Ali et al., 2022; Du et al., 2022). In the metal leaching, various types of inorganic acids (HCl, HNO_3_, H_2_SO_4_, and H_3_PO_4_) (Botelho Junior et al., 2021) or alkaline (e.g., NaOH) solutions are employed (Mao et al., 2022). Takahashi et al. (2020) investigated the leaching of Co from spent LIBs obtained from a cell phone company using various inorganic acid leaching agents [H_2_SO_4_, HNO_3_, and HCl with or without reducing agent (H_2_O_2_)]. They found that the acid leaching using a combination of H_2_SO_4_ and H_2_O_2_ at the solution pH of 3.0 and temperature of 50°C resulted in the best Co recovery (98%). Another recent study from the same research group on metal recovery from the spent NMC type battery using 1 mol/L H_2_SO_4_ at the temperature of 90°C and solid-to-liquid ratio of 1:10, but without addition of a reducing agent achieved 100% extraction of Co, Li and Ni and 93% extraction of Mn (Guimarães et al., 2022). The three key steps of the hydrometallurgical method include leaching, precipitation and solvent extraction (Hua et al., 2020). Among the pyrometallurgical and hydrometallurgical processes, hydrometallurgical process is more advantageous because of less greenhouse gases (GHGs) (e.g., CO_2_) emissions and low energy consumption (Vasconcelos et al., 2023). Additionally, hydrometallurgical processing results in the recovery of highly pure-grade Li. However, the above recycling methods (pyrometallurgy and hydrometallurgy) are not sustainable since the pyrometallurgical method is energy intensive and produces GHGs (Ali et al., 2022). Although the GHGs emission rate is lower in hydrometallurgical than pyrometallurgical method, it produces a high amount of corrosive wastewater which could damage the receiving water bodies if discharged without proper treatment (Ali et al., 2022).

In the hydrometallurgical process, H_2_O_2_ is usually used as a reducing agent for metal extraction from spent batteries using an inorganic-based leaching system (Takahashi et al., 2020). However, H_2_O_2_ is a chemical reagent that is explosive in nature and can easily disintegrate in the acidic (e.g., H_2_SO_4_) condition (Wu et al., 2020; Ma et al., 2021). Thus, for the development of environmentally friendly hydrometallurgical processes, considerable interest has emerged in the use of green reductants [e.g., antibiotic bacteria residues (ABR) and fruit peel] while using hydrometallurgical-based leaching process (Wu et al., 2020; Ma et al., 2021). In H_2_SO_4_ leaching system at the liquid-to-solid ratio of 30:1 ml/g, temperature of 90°C and reaction time of 2.5 h, the application of ABR (ABR to spent cathode powder: 0.8:1) resulted in the optimum recovery of various metals namely Co (98.50%), Li (99.90%), Ni (99.57%) and Mn (98.99%) (Ma et al., 2021). A subsequent study was conducted by the same research group in which the authors initially conducted thermal (350–750°C) reductive transformation of spent cathode powder in the presence of ABR (Ma et al., 2022). The highest recovery of various metals (Co: 99.5%, Li: 99.9%, Ni: 99.4%, and Mn: 99.9%) was obtained under low concentration acid leaching conditions (1 mol/L H_2_SO_4_) with a liquid-to-solid ratio of 20:1, reaction temperature of 60°C and reaction time of 1 h. Wu et al. (2020) used waste orange peel as the green reductant for metal extraction from spent LIBs. Citric acid-based leaching (1.5 M) with the orange peel dose of 5 mg/ml at the reaction temperature of 100°C, reaction duration of 4 h, and slurry density of 25 g/mL was effective for removal of various metals (Co, Li, Ni, and Mn), i.e., the leaching efficiency of metals varied between 80 and 99%.

The direct recycling is reported to be economically feasible with no considerable negative effects on the environment and energy efficient (Ali et al., 2022; Roy et al., 2022). Practically it is feasible to recover all battery materials including anodes, foils and electrolytes, and the process is most suitable for LFP type batteries (Roy et al., 2022). However, the key disadvantages are: (1) the maturity level of process is low (mainly at the laboratory scale, i.e., will take time to mature and commercialize), (2) the regeneration process is yet to be developed and (3) the mixing of cathode materials decreases the performance and value of the recycled products (Ali et al., 2022; Roy et al., 2022).

**FIGURE 3:**
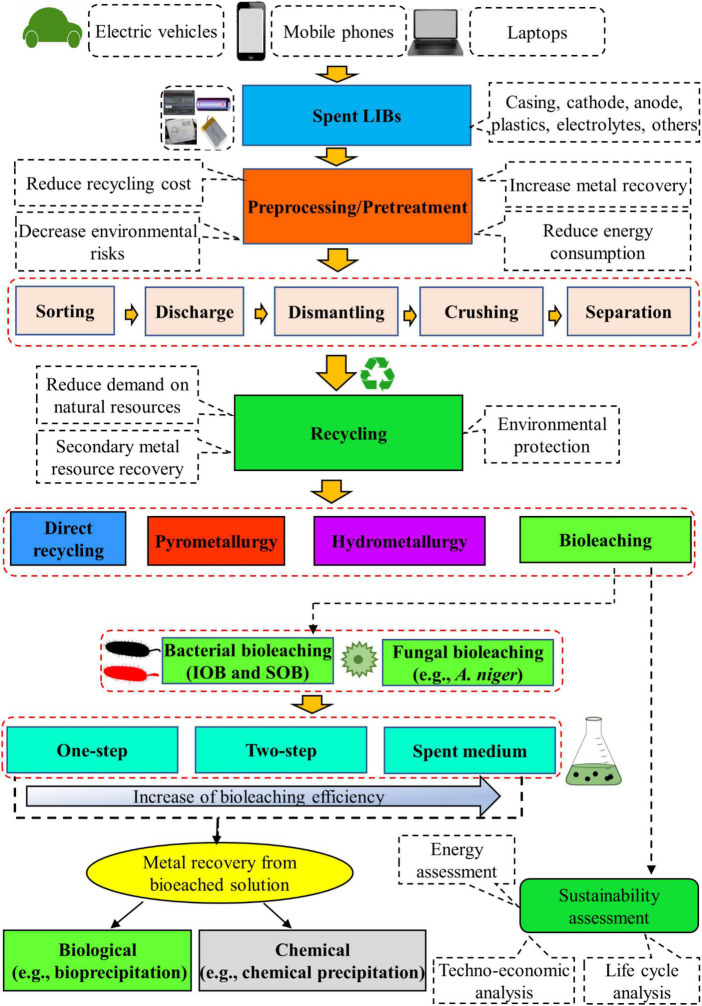
Flow-chart for recycling of spent LIBs.

In recent years, increasing interests are given on the application of bio-hydrometallurgical (bioleaching) methods which use acid producing microorganisms (bacteria or fungi) for the recovery of valuable metals from spent LIB since the bleaching process is cost-effective, environmentally friendly, less energy intensive, and has low emissions of GHGs as well as high efficiency (Hua et al., 2020; Roy et al., 2021a; Du et al., 2022). Other potential advantages include the requirement of minimal chemicals and water for bioleaching process, operational simplicity, no need of high skilled workers, selectivity toward metals, growth of most of the microbes under ambient conditions, and continued reuse of microbes (Roy et al., 2021a; Golmohammadzadeh et al., 2022; Ratnam et al., 2022). Microbial adaptation to toxic environments, genetic engineering of microbes using synthetic biology techniques, bioprospecting of novel biomining bacteria and storing of microbial agents have enhanced the accessibility of appropriate biocatalysts for applications in bioleaching (Kaksonen et al., 2018, 2020). Furthermore, the development of advanced microbial characterization tools has improved the understanding of metabolisms and metabolic activities of microbial communities and their abilities in the bioprocesses. Bioleaching has already been commercially applied for the removal of metals from low-grade sulfidic ores and for the pretreatment of refractory sulfidic gold-containing minerals (Kaksonen et al., 2020). At present, the application of bioleaching techniques for the recycling of toxic and complex waste materials such as spent LIBs have received considerable attention. However, the bioleaching process is slower than the hydrometallurgical process (Botelho Junior et al., 2021). The detailed information about the recovery of valuable metals from spent LIBs using bioleaching is presented in the next section. Additionally, a flow diagram containing the detailed procedures involved on the recycling of spent LIBs using bioleaching method is presented in Figure 3.

## 5. Microbial-based (bioleaching) recovery of valuable metals from spent LIBs

Bioleaching is a microbial-based natural chemical process in which insoluble solids are converted to soluble and extracted forms (Villares et al., 2016; Heydarian et al., 2018). Moreover, bioleaching is a promising emerging biotechnological process for recovery of secondary metal resources from spent LIBs, and could also contribute to achievement of the environmentally friendly circular economy (Orell et al., 2010; Villares et al., 2016). The bioleaching experiments are mainly conducted in three different ways namely (1) one-step bioleaching, (2) two-step bioleaching and (3) spent-medium bioleaching based on the types of interactions (direct/indirect) between microorganisms and the pre-processed spent LIBs (crushed and sieved powder form of spent LIBs) (Horeh et al., 2016; Biswal et al., 2018, 2022). The one-step method is a conventional approach in which pre-growth microorganisms are added as an inoculum to the leaching medium containing the spent LIBs powder. The leaching of metals from the complex spent LIBs powder matrix is driven by the continuous production of bioacids with the concurrent microbial growth (Biswal et al., 2022). The one-step method is suitable for spent LIBs containing a low amount of toxic components, e.g., pre-processed LIBs (e.g., water washing and drying) since the growth rate of microorganisms would be reduced due to toxicity effects. In the two-step method, the leaching organism is primarily grown (e.g., up to logarithmic phase) in the leaching medium for a specific time period for the production of bioacids. The LIB powered is then added to initiate the metal extraction process (Biswal et al., 2018, 2022). This method is suitable for spent LIBs containing toxic materials (without having any pre-treatments like washing and drying) which could hinder the growth of microorganisms with direct application. In both one-step and two-step methods, direct physical contact/interaction between microorganisms and spent LIB components occurs. In the spent medium method, the leaching is carried out by adding battery powder to the cell free medium (called spent medium), i.e., the microorganism is firstly fully grown in the medium for the production of bioacids, then the spent medium is obtained by removing the cells using various techniques such as centrifugation, filtration, or both (Biswal et al., 2018, 2022). The spent medium-based bioleaching can be applied to the spent LIBs containing diverse toxic components namely metals, electrolytes and organic solvents. Among the three types of bioleaching approaches, the spent medium bioleaching is most preferable since the leaching efficiency is usually higher in spent medium-based bioleaching tests compared to others (one-step or two-step method) (Horeh et al., 2016; Alavi et al., 2021; Lobos et al., 2021). Since there is no physical contact between microbial agents and spent LIBs particles in spent medium leaching, the individual process (biological and chemical) can be optimized (Bahaloo-Horeh et al., 2019). For example, the quality and quantity of biogenic acids can be enhanced during the initial growth experiments, while in the chemical leaching, the use of biogenic acid and higher pulp density can be explored since there is no issue of toxicity effects on hazardous components of LIBs due to absence of microbial cells. The bacterial-based leaching is conducted using basal salt/9K/modified 9k medium [(NH_4_)_2_SO_4_, KCl, K_2_HPO_4_.3H_2_O, MgSO_4_.7(H_2_O), Ca(NO_3_)_2_, and FeSO_4_.7(H_2_O)] (Roy et al., 2021b) while fungal leaching is carried out using the sucrose medium [(NaNO_3_, KH_2_PO_4_, KCl, MgSO_4_.7H_2_O), yeast extract and sucrose] (Bahaloo-Horeh and Mousavi, 2017). The bioleaching experiments are conducted using single/pure culture microbial systems (Biswal et al., 2018) or consortia/mixed culture microbial systems (Heydarian et al., 2018; Alavi et al., 2021). The initial inoculum size for bacterial and fungal leaching is nearly 10^7^ cells/spores per mL (Mishra et al., 2008; Bahaloo-Horeh and Mousavi, 2017).

### 5.1. Bacterial-based bioleaching for recovery of valuable metals from spent LIBs

The acidophilic sulfur-oxidizing bacteria (SOB) and iron-oxidizing bacteria (IOB) are widely used for the bioleaching of valuable metals from spent LIBs (Ghassa et al., 2020; Noruzi et al., 2022). The major SOB employed in the bioleaching of valuable metals from spent LIBs include *Acidithiobacillus thiooxidans*, *Sulfobacillus thermosulfidooxidans*, and *Alicyclobacillus* spp. while the dominant IOB applied for spent LIB bioleaching are *Acidithiobacillus ferrooxidans*, *Leptospirillum ferriphilum*, and *Sulfobacillus* spp. (Ghassa et al., 2020; Liu et al., 2020; Golmohammadzadeh et al., 2022). These SOB and IOB are called chemolithoautotrophs which can utilize carbon dioxide (CO_2_) as the carbon source (i.e., they obtain carbon by reductive fixation of atmospheric CO_2_), while they utilize inorganic compounds such as ferrous ion (Fe^2+^, IOB) and reduced S [elements sulfur (S^0^), SOB] as an energy source (Hong and Valix, 2014). In the bacterial leaching, ferrous sulfate (FeSO_4_), iron powder and pyrite (FeS_2_) are also used as the source of iron and sulfur (Golmohammadzadeh et al., 2022). The IOB oxidizes Fe^2+^ to Fe^3+^, while SOB oxidizes S^0^ to SO_4_^2–^ (Heydarian et al., 2018). Other chemolithoautotrophic bacteria namely *Acidithiobacillus caldus*, and *Ferroplasma* spp. are also applied for the bioleaching of metals from spent LIBs (Ghassa et al., 2020). A majority of the chemolithotrophic bacteria show high level of tolerance to metals toxicity (Işıldar et al., 2019).

Single or consortia acidophilic bacteria are used for the recovery of valuable metals (mainly Co and Li) from spent LIBs (Table 4). Biswal et al. (2018) used *A. thiooxidans* 80191 as the microbial agent and observed higher Li (66%) removal than Co (23%) under two-step bioleaching tests. Another study also used *A. thiooxidans* (PTCC 1717) (inoculum concentration: 10^7^ cells/mL) for the metal extraction from spent LIBs (spent coin cells) in two-step bioleaching tests, and found higher recovery of Co (60%) and Li (99%) than Mn (20%) (Naseri et al., 2019b). Using an IOB, *A. ferrooxidans*, Roy et al. (2021b) obtained higher Co (94%) recovery compared to Li (60%) from spent LIBs at a high pulp density (100 g/L) in 3 days with continued refilling of the bacterial culture to the leaching medium for three cycles. Additional study from the same research group on the spent nickel-, manganese-, cobalt (NMC)-based LIBs using *A. ferrooxidans* at a higher pulp density (100 g/L) reported a higher extraction efficiency of various metals namely Co (82%), Li (89%), Mn (92%) and Ni (90%) (Jegan-Roy et al., 2021). Li et al. (2013) reported 47.6% dissolution of Co from spent LIB employing *A. ferrooxidans* as the leaching organism. Using the mixed culture consortia of *A. ferrooxidans* and *A. thiooxidans*, 67% Co and 80% Li were extracted from spent LIBs in the nutrient rich medium (Marcinčáková et al., 2016). However, the metal efficiency was considerably reduced (10.5% Co and 35% Li) when tested with the low nutrient medium which contains only elemental sulfur (4 g/L) and sulphuric acid. Another study also used the same mixed culture (*A. ferrooxidans* and *A. thiooxidans*), and found 50.4 % Co, 99.9% Li and 89.4% Ni recovery from spent LIBs used in laptops (Heydarian et al., 2018). However, using a mixed culture containing four different thermophilic bacteria (*A. caldus*, *L. ferriphilum*, *Sulfobacillus* spp. and *Ferroplasma* spp.), the bioleaching tests resulted in the dissolution of 99.9% Co, 84% Li and 99.7% Ni from spent LIB used in laptops. Do et al. (2022) performed spent NMC bioleaching using *A. ferrooxidans*, and reported 90.4% Co, 89.9% Li, 85.5% Ni, 91.8% Mn recovery in 6 h at a higher pulp density (100 g/L). Additionally, the authors attempted to regenerate cathode material (NMC_111_ and NMC_622_) using the oxalate-based precipitated metals from the bioleached solution and found that the electrochemical stability of the regenerated cathode material was similar to that of commercial NMC (i.e., nearly 85% of capacity retention after 50 cycles at 100 mA/g). Liu et al. (2020) reported that bio-oxidative activity of microbial agents reduced due to metallic stress, as a result, the bioleaching efficiency is declined. However, addition of exogenous glutathione (GSH) which is a ubiquitous intracellular peptide with diverse functions (0.3 g/L), the bacterial intracellular reactive oxygen species (ROS) level decreased by 40% which resulted in 96.3% Co and 98.1% Li recovery at pulp density of 5% using microbial consortium of *L. ferriphilum* and *S. thermosulfidooxidans*.

**TABLE 4:** Bacterial-based bioleaching for recovery of valuable metals from spent Li-ion batteries (LIBs).

		Bioleaching efficiency				
**Bacteria**	**Key leaching condition**	**Co**	**Li**	**Other metals**	**Additional information**	**References**
*A. thiooxidans* (80191)	Pulp density: 0.25% (w/v), pH: 2.4	23%	60%	NA	Co and Li dissolution were higher in two-step bioleaching	Biswal et al., 2018
*A. ferrooxidans* (ATCC 19859)	Solid-to-liquid ratio: 5 g/L, pH: 2.5	65%	9.5%	NA	Higher solid/liquid ratios reduced leaching efficiency	Mishra et al., 2008
*A. ferrooxidans* (DSMZ, 1927)	Pulp density: 100 g/L	94%	60.30%	NA	For optimum metal extraction, replenishment of microbial culture was done every 24 h for 3 cycles	Roy et al., 2021b
*A. ferrooxidans* (DSMZ 1927)	Pulp density: 100 g/L	82%	89%	Mn: 92%, Ni: 90%	Leaching efficiency was increased with increase of sulphuric and ferric ion in the leaching medium as well as by replenishing the culture for three cycles	Jegan-Roy et al., 2021
*A. ferrooxidans* (DSMZ 1927)	Pulp density: 100 g/L	90.4%	89.9%	Mn: 91.8%, Ni: 85.5%	NMC (NMC_111_ and NMC_622_) were regenerated from the oxalate-based coprecipitated product. The electrochemical stability of the regenerated NMC was similar to the commercial NMC.	Do et al., 2022
*A. ferrooxidans* (isolated)	Pulp density: 1% (s/v), bacteria inoculation: 5% (v/v), pH: 1.5	47.60%	NA	NA	Enhancement of cobalt dissolution was observed at higher redox potential	Li et al., 2013
*A. thiooxidans* (PTCC 1717)	Pulp density: 30 g/L, pH: 2.0	60%	99%	Mn: 20%	Bioleached spent LIB residue was safe to disposal as meets the TCLP limit	Naseri et al., 2019b
*A. thiooxidans* (PTCC 1647)	Pulp density: 40 g/L, pH: 2.0	88%	100%	Mn: 20%	The shrinking core model predicted that the diffusion of ferric ions plays a key role in metal leaching.	Naseri et al., 2019a
*A. ferrooxidans* (isolated)	Pulp density 1% (s/v)	99.90%	NA	NA	Enhancement of cobalt dissolution was noticed with addition of copper ions (0.75 g/L).	Zeng et al., 2012
*A. ferrooxidans* (isolated)	Pulp density 1% (s/v)	98.40%	NA	NA	Enhancement of cobalt dissolution was noticed with addition of silver ions (0.02 g/L).	Zeng et al., 2013
*A. ferrooxidans* (PTCC 1647)	Pulp density: 10 g/L	19.0%	67%	Mn: 50%, Ni: 34%	Ultrasonic treatment (203.5 W for 30 min) enhanced metal leaching efficiency.	Nazerian et al., 2023
*A. ferrooxidans* (isolated)	pH: 2.5, inoculum concentration: 20% (v/v)	57.8%	NA	NA	Highest Co recovery was found at an inoculum concentration of 20% (v/v) in 14 days of incubation time.	Hariyadi et al., 2022b
*A. ferrooxidans* (isolated)	Pulp density: 10 g/L, pH: 2- 4, inoculum concentration: 20% (v/v)	73.95%	NA	NA	Bacterial strain isolated from the acid mine drainage has the potential as oxidizing agent for recovery of metals (Co and Li) from spent LIBs.	Putra et al., 2022
*L. ferriphilum* (isolated)	Pulp density: 1% (w/v), pH: 1.0	NA	49%	NA	Leaching tests were done using pyrite (FeS_2_, 16 g/L) as the energy source and LFP as the cathode material.	Xin et al., 2016
*A. thiooxidans* (isolated)	Pulp density: 1% (w/v), pH: 1.0	NA	98%	NA	Leaching tests were done using S^0^ (16 g/L) as the energy source and LFP as the cathode material.	Xin et al., 2016
*A. thiooxidans* (isolated)	Pulp density: 1% (w/v), pH: 1.0,	NA	97%	Mn: 35%	Leaching tests were done using S^0^ (16 g/L) as the energy source and LMO as the cathode material.	Xin et al., 2016
Mixed bacterial culture (isolated)	Pulp density: 2 g/L, pH: 7.0	NA	63.8%	NA	Adaptation of bacteria with LiCl solution (576 μM) enhanced leaching efficiency of bacteria to Li.	Hartono et al., 2017
Mixed culture of IOB and SOB (isolated)	Pulp density 10 g/L, pH: 1.5 (2.0 g/L sulfur + 2.0 g/L FeS_2_)	90.00%	80.0%	NA	Acidolysis was the main mechanism for Li dissolution, whereas both acidolysis and redoxolysis contributed for Co dissolution.	Xin et al., 2009
Mixed culture 1	Iron sulfate: 36.7 g/L; sulfur: 5.0 g/L, pH: 1.5	50.40%	99.20%	Ni: 89.4%	Metal contents in spent LIB residue reduced to below the regulatory standard (USEPA), thus the bioleached LIBs can be reused or disposed safely	Heydarian et al., 2018
Mixed culture 1	Pulp density: 1% (w/v), pH: 2.0	99.95%	NA	Ni: 99.95%	High metal extraction yield observed in short time (3 days) in two-step leaching with addition of silver ions (0.02 g/L)	Noruzi et al., 2022
Mixed culture 1	Pulp density: 10% (w/v), pH: 1.8	53.20%	60.00%	Ni: 48.7%, Mn: 81.8%, Cu: 74.4%	Biogenic ferric ion-based critical metal leaching yield was further improved with addition 100 mM H_2_SO_4_.	Boxall et al., 2018
Mixed culture 2	Pulp density: 10 g/L, pH: 1.8	99.90%	84%	NA	Similar leaching results obtained by using iron scrap waste instead of chemical reagent (FeSO_4_.7H_2_O)	Ghassa et al., 2020
Mixed culture 1	Pulp density: 10 g/L, pH: 1.5	67%	80%	NA	Leaching efficiency was reduced to 35% Li and 10.5% Co in low nutrient medium	Marcinčáková et al., 2016
Mixed culture 3	Pulp density: 5% (w/v), pH: 1.25	96.3%	98.1%	NA	Reactive oxygen species (ROS) regulation by the exogenous addition of glutathione resulted higher metal leaching yield.	Liu et al., 2020
Mixed culture 4	Pulp density: 1% (w/v), pH: 1.0	96.0%	92.0%	Mn: 92%, Ni: 97%	The experiments were conducted using mixed culture and mixed energy source (S^0^ + FeS_2_) and NMC as cathode material	Xin et al., 2016
Mixed culture 4	Pulp density: 1% (w/v), pH: 1.0	NA	> 95%	Mn: > 95%	The experiments were conducted using mixed culture and mixed energy source (S^0^ + FeS_2_) and LMO as cathode material	Xin et al., 2016
Mixed culture 5	Pulp density: 2% (w/v), pH: 1.0	72.0%	89.0%	NA	Thermodynamics analysis shows bioleaching has much greater potential to happen compared to chemical leaching.	Niu et al., 2014
Mixed culture 6	Pulp density: 15 g/L, pH: 1.20	99.3%	100%	NA	Extracellular polymeric substances (EPS) secreted by bacteria enhanced metal removal.	Wu W. et al., 2019

Mixed culture 1: *A. ferrooxidans* and *A. thiooxidans*. Mixed culture 2: *A. caldus, L. ferriphilum, Sulfobacillus* spp. and *Ferroplasma* spp. Mixed culture 3: *L. ferriphilum* and *Sulfobacillus thermosulfidooxidans*. Mixed culture 4: *A. thiooxidans* and *L. ferriphilum*. Mixed culture 5: *Alicyclobacillus* spp. and *Sulfobacillus* spp. Mixed culture 6: *L. ferriphilum* spp. and *S. thermosulfidooxidans* spp. *A. thiooxidans, Acidithiobacillus thiooxidans; L. ferriphilum, Leptospirillum ferriphilum*; NA, absence of data.

A few studies have isolated acidophilic bacteria from the acid mine drainage, then applied them for metal extraction from spent LIBs (Hariyadi et al., 2022b; Putra et al., 2022). Hariyadi et al. (2022b) conducted bioleaching of spent LIBs using *A. ferrooxidans* cells isolated from water samples of a coal mine pond, and found recovery of 57.81% Co at medium pH of 2.5 in 14 days of incubation period. Another study from the same research group reported 73.95% dissolution of Co from spent LIBs at an optimum experimental condition of 10 g/L of pulp density, temperature of 30°C, pH of 2–4 and incubation period of 14 days with *A. ferrooxidans* inoculum size of 20% (v/v) (Putra et al., 2022). Overall, findings of these reports suggest that the metal removal efficiency varied among various studies which may be due to the difference in the leaching microorganisms (e.g., single vs. mixed culture), mode of leaching (one-step, two-step or spent medium), battery chemistry (e.g., characteristics of cathode materials) and leaching conditions (pH and pulp density). Additionally, in most of the studies, it was observed that the Li bioleaching efficiency was greater than that of Co.

### 5.2. Fungal-based bioleaching for recovery of valuable metals from spent LIBs

Fungi are the heterotrophic microorganisms which use organic carbon-based materials as the carbon source for their growth and metabolism (Bahaloo-Horeh et al., 2019). Several fungal species namely *Aspergillus niger*, *Aspergillus tubingensis*, *Penicillium simplicissimum*, and *Penicillium chrysogenum* are employed for the extraction of metals from electronic wastes (Bahaloo-Horeh et al., 2019; Işıldar et al., 2019; Lobos et al., 2021). However, in spent LIBs bioleaching, *A. niger* is highly favored due to less complexity in the growth and harvesting process and higher yields (Roy et al., 2021a). In contrast to bacteria, fungi have the greater capacity for tolerance to diverse toxic metals, having a shorter lag phase and faster leaching rate as well as fungi can grow in both acid- and alkaline-consuming wastes (Horeh et al., 2016). A study compared the metal tolerance capacity of three fungi species namely *A. niger* (ATCC 6275), *P. chrysogenum* (ATCC 10108) and *P. simplicissimum* (ATCC 48705) by exposing them 250 mg/L of CoCl_2_ or LiCl solution over a period of 20 days (Lobos et al., 2021). Among the three fungal species, only *A. niger* developed tolerance to both metals since an increase of biomass production was observed.

Biswal et al. (2018) conducted fungal bioleaching of spent LIBs (pulp density: 0.25% w/v) using two isolated strains, *A. niger* MM1 and *A. niger* SG1 under cell-free spent medium. Both fungal strains were effective for the extraction of valuable metals from spent LIBs, i.e., 80–82% Co and 100% Li recovery were achieved. Horeh et al. (2016) used a pure culture of *A. niger* (PTCC 5210) for the recovery of various metals from spent mobile phone LIBs using three different approaches (one-step, two-step and cell-free spent medium). Among the three types of experimental conditions, the spent-free medium test exhibited highest performance for the extraction of numerous valuable metals including Co (45%), Li (95%) and other metals (e.g., Cu: 100%, Mn: 70%, and Al: 65%). Subsequent experiments from the same research group using *A. niger* (PTCC 5210) reported the highest Co (64%) and Ni (54%) recovery at lower (1%, w/v) pulp density, but the recovery of other four metals (Cu: 100%, Li: 100%, Mn: 77% and Al: 75%) was optimum at higher (2%, w/v) pulp density (Bahaloo-Horeh and Mousavi, 2017).

A recent study isolated *A. niger* from waste spices (Candlenut), and 57% Co and 72% Li recovery was obtained in 21 days of incubation time (Hariyadi et al., 2022a). Using a mixed fungal culture (*A. niger* and *A. tubingensis*), Alavi et al. (2021) investigated the bioleaching of valuable metals from spent cellphone LIBs using three different types of carbon sources (pure sucrose, impure sucrose and vinasse from an ethanol industry) under three types of leaching methods. The bioleaching was optimum with the spent medium test having vinasse as the carbon source, i.e., the recovery of Co and Li was nearly 60 and 95%, respectively, whereas the recovery of another three metals (Mn, Ni, and Al) was varied between ∼80 and 98%. To enhance the metals toxicity tolerance level of *A. niger* (PTCC 5010), Bahaloo-Horeh et al. (2018) gradually increased the pulp density in the leaching medium from 0.3 to 1.0% (w/v) to adopt the *A. niger* to the toxic metal environment. Bioleaching tests showed that the adapted *A. niger* exhibited higher leaching efficacy for diverse metal elements from mobile phone-based spent LIBs including Co (38%), Li (100%), Cu (94%), Mn (72%), Al (62%), and Ni (45%). A study compared the metal dissolution performance of two different types of fungal species [*A. niger* (PTCC 5010) and *P. chrysogenum* (PTCC 5037)] (Kazemian et al., 2020), and the authors noticed that the *A. niger* (76.31%) demonstrated higher Li leaching capability than *P. chrysogenum* (54.6%). In total, most of the studies used *A. niger* as the microbial agent for the fungal bioleaching of spent LIBs (Table 5). Among various fungal species, *A. niger* is capable of tolerating metals toxicity from spent LIBs, and hence exhibited higher metal bioleaching efficiency. Like bacterial-based bioleaching, Li recovery was higher than Co in fungal-based bioleaching.

**TABLE 5:** Fungal-based bioleaching for recovery of valuable metals from spent Li-ion batteries (LIBs).

		Metal bioleaching efficiency				
**Fungal**	**Key leaching condition**	**Co**	**Li**	**Other metals**	**Additional information**	**References**
*Aspergillus niger* (PTCC 5210)	Pulp density: 1% (w/v); pH: 6.0	45%	95%	Cu: 100%, Mn: 70%, Al: 65%, Ni: 38%	Spent medium exhibited highest metal extraction yield. Also, bioacids yielded higher metal extraction than synthetic chemical acids.	Horeh et al., 2016
*A. niger* MM1/SG1 (isolated)	Pulp density: 0.25% (w/v); carbon source: sucrose; pH: 3.5	80–82%	100%	NA	Leaching efficiency was higher in cell-free spent medium. Also, bioacids yielded higher metal extraction than the synthetic chemical acid (citric acid).	Biswal et al., 2018
*A. niger* (PTCC 5210)	Pulp density: 1–2% (w/v), carbon source: sucrose	64%	100%	Cu: 100%, Mn: 77%, Al: 75%, Ni: 54%	Co and Ni recovery were higher at 1% pulp density, while Li, Cu, Al and Mn recovery was higher at 2% pulp density.	Bahaloo-Horeh and Mousavi, 2017
*A. niger* (PTCC 5210)	Pulp density: 1 % (w/v), carbon source: sucrose	38%	100%	Cu: 94%, Mn: 72%, Al: 62%, Ni: 45%	Adapted fungi showed higher metal leaching performance compared to unadopted fungi.	Bahaloo-Horeh et al., 2018
*A. niger* (PTCC 5010)	Pulp density: 10% (w/v), carbon source: glucose; pH: 4.5,	NA	73.3%	NA	*A. niger* showed higher metal leaching performance than *Penicillium chrysogenum*.	Kazemian et al., 2020
*P. chrysogenum* (PTCC 5037)	Pulp density: 10% (w/v), carbon source: glucose; pH: 4.5,	NA	54.6%	NA	*A. niger* showed higher metal leaching performance than *P. chrysogenum*.	Kazemian et al., 2020
*A. niger* (isolated)	Carbon sources: glucose, incubation time: 21 days	57%	72%	NA	Highest valuable metal recovery obtained in the one step process	Hariyadi et al., 2022a
Mixed culture 1	Pulp density: 1% (w/v), carbon source: sucrose, impure sucrose or vinasse	∼60%	∼95%	Mn: ∼98%, Ni: ∼80%, Al: ∼82%	Spent medium leaching showed higher metal recovery efficiency with vanasse as the carbon source	Alavi et al., 2021

Mixed culture 1: *A. niger* and *Aspergillus tubingensis*. NA, absence of data.

### 5.3. Quality and quantity of bioacids production in bacterial and fungal bioleaching

Bioacids or biogenic acids are the metabolites produced by the microbial agents during their growth in the leaching medium with or without supplementation of spent LIBs, and they primarily contribute to the extraction of metals from the spent LIB solid matrices (Biswal et al., 2018, 2022). In bacterial-based leaching [specifically SOB (e.g., *A. thiooxidans*)] with the use of elemental sulfur as the energy source and electron donor, biogenic sulfuric acid (H_2_SO_4_) is produced by oxidation of S^0^ (Biswal et al., 2018). With the use of *A. thiooxidans* (80191), the pure culture growth medium resulted in the production of 10.2 mM biogenic H_2_SO_4_, while the H_2_SO_4_ production significantly decreased by nearly fivefold (1.7 mM) with the addition of 1% (w/v) S*^o^* as the energy source and 0.25% (w/v) spent LIB powder (one-step leaching) (Biswal et al., 2018). Using the *A. ferrooxidans* strain (DSMZ, 1927), Roy et al. (2021b) reported 0.17 M production of H_2_SO_4_ under the leaching condition of 100 mg/L spent LIB pulp density and 45 g/L FeSO_4_. However, the H_2_SO_4_ concentration increased by nearly 3 times (0.52 M) with the increase of FeSO_4_ dose to 150 g/L.

In fungal bioleaching, mostly sucrose and glucose are used as a carbon and energy source, and the microbial metabolism (through the Krebs cycle) results in the production of diverse organic acids (carboxylic acids) namely citric, gluconic, oxalic, malic, fumaric, lactic, pyruvic and succinic acids, etc (Bahaloo-Horeh and Mousavi, 2017; Bahaloo-Horeh et al., 2019). Using two isolated *A. niger* strains (MM1 and SG1), Biswal et al. (2018) reported only the production of citric acid with 76.9–102.4 mM in the pure culture medium (without spent LIBs), but the citric acid concentration was reduced to nearly half (40.7–43.1 mM) with the one-step bioleaching. Similar observations were also reported by two earlier bioleaching works since the citric acid was the dominant metabolite produced by the metabolism of sucrose, and the concentration of citric acid is usually lower in one-step/two-step bioleaching (133 mg/L) compared to cell free spent medium leaching (8,078 mg/L) (Horeh et al., 2016; Bahaloo-Horeh and Mousavi, 2017). Kim et al. (2016) found that *A. niger* isolate (KUC5254) produced a significant amount of citric acid (118.8 mM) than oxalic acid (0.8 mM) in the sucrose (100 g/L) growth medium.

A study compared the changes of quality of organic acid production by the unadapted and adapted *A. niger* (adapted by adding various doses of spent LIBs pulp densities), and only oxalic acid (up to 13,000 mg/L) was produced by the unadapted *A. niger*, whereas four different organic acids (oxalic, malic, citric and gluconic acid) were produced by the adapted *A. niger* with gluconic acid was the dominant metabolite (nearly 4,000–13,000 mg/L) with incubation time varied between 6 and 30 days (Bahaloo-Horeh et al., 2018). Overall, the quantity and quantity of bioacids production depends on the various factors namely the type of microbial agents (bacteria vs. fungi), leaching medium chemistry (e.g., composition and pH), type of energy/carbon sources, spent LIBs characteristics (e.g., quantity and quality of metals), pulp density, etc (Supplementary Table 2) (Biswal et al., 2018, 2022; Bahaloo-Horeh et al., 2019). The concentration of bioacids is usually higher in the pure culture growth medium (i.e., absence of spent LIBs) than that of the microbial growth in the presence of waste materials (one-step or two-step leaching) (Biswal et al., 2018). The decrease in the generation of bioacids by the addition of spent LIBs could be due to deactivation/suppression of enzyme activities responsible for the bioacid production by the toxic/inhibitory effects of metals or other components of spent LIBs (Naseri et al., 2022; Pourhossein and Mousavi, 2023).

### 5.4. Comparison of valuable metal recovery efficiency between bacterial, fungal and chemical leaching

A few studies compared the performance of valuable metals recovery of two types of biological leaching processes (bacteria vs. fungi) as well as the leaching efficiency between bioleaching and chemical leaching (i.e., using commercially synthesized chemical acids with concentrations similar to the concentration of bioacids produced in bioleaching) (Bahaloo-Horeh et al., 2018; Biswal et al., 2018; Noruzi et al., 2022). According to Biswal et al. (2018) between bacterial and fungal bioleaching, the metal extraction efficiency from spent LIBs was higher in fungal leaching (Co: 82% and Li: 100%) compared to bacterial leaching (Co: 23% and Li: 66%). Additional experiments using commercially synthesized of H_2_SO_4_ and citric acid with concentrations similar to those of bioacids showed that the percentage of valuable metal dissolution was higher (4–15%) in bioleaching compared to chemical reagent-assisted leaching method. Similar results were also obtained by another study in which authors compared critical metals removal efficacy between fungal leaching and chemical leaching (employing a mixture of commercial four types of carboxylic acids namely citric, gluconic, malic and oxalic acids) (Bahaloo-Horeh et al., 2018). The metal removal efficiency was higher in fungal-based leaching (38% Co and 100% Li) compared to commercial organic acid-based leaching (13% Co and 68% Li). According to a recent study, the recovery of Co and Ni from spent LIBs was higher in bacterial-based leaching (99.95% for both Co and Ni using *A. ferrooxidans* and *A. thiooxidans*) than chemical leaching [only 7.09% Co and 26.90% Ni using Fe_2_(SO_4_)_3_ with the sulfate concentration similar to that in the bioleaching solution] (Noruzi et al., 2022). Xin et al. (2009) reported that the chemical simulation of acid solubilization (Fe^2+^: 4 g/L and H_2_SO_4_ at pH of 1.0) resulted 621 mg/L Co and 303 mg/L Li recovery from spent LIBs powder. However, the bioacid leaching system achieved much higher metal dissolution (i.e., Co: 920 mg/L and Li: 470 mg/L) which is potentially due to continuous production of H_2_SO_4_ from biooxidation of S^0^ by SOB. Additional work from the same research group on three types electric vehicle spent cathode materials (LMO, LFP, and NMC) also found that the metal dissolution was higher in bioleaching system containing mixed bacterial culture (*A. thiooxidans* and *L. ferriphilum*) and mixed energy source (S^0^ and FeS_2_), i.e., leaching of various metals in the bioacid system was 92% Li, 43.5% Co, 92% Mn, and 38.3% Ni, whereas the leaching of these metals in chemical simulation system was much lower, i.e., 65, 20, 52, and 18%, respectively.

Thermodynamics analysis shows that bioleaching is more thermodynamically feasible than chemical leaching (Niu et al., 2014). Niu et al. (2014) performed the thermodynamic analysis of the bacterial leaching (mixed culture of *Alicyclobacillus* spp. and *Sulfobacillus* spp.) and chemical leaching (H_2_SO_4_ + FeSO_4_), and found that the change of free energy (ΔG) for the bacterial leaching (−3629.93 KJ/mol) was nearly 14 times higher than that of the chemical leaching (ΔG: −265.44 KJ/mol). The large difference in the free energy value between the two types of leaching suggests that the bioleaching has much higher potential to be a favorable compared to chemical leaching. In fungal bioleaching, multiple metabolites (organic acids) are produced, while in bacterial-based bioleaching only one metabolite (H_2_SO_4_) is produced. Thus, it is expected that higher metal recovery is possible by the chemical action of multiple metabolites in fungal leaching than bacterial leaching with single metabolite. The bioacids (carboxylic acids) produced in fungal leaching are mild, less toxic and biodegradable, while bacterial leaching produces inorganic acids such as H_2_SO_4_ which are corrosive and not easy to handle (Bahaloo-Horeh and Mousavi, 2017). Based on the pKa values of carboxylic acids, the pH of dilute solutions of carboxylic acids (equivalent to the fungal produced bioacids) is in the moderate acidic pH range between 3 and 5 (Moosakazemi et al., 2022). Sedlakova-Kadukova et al. (2020) compared the performance of three different bioleaching systems (bacterial consortia: *A. ferrooxidans* and *A. thiooxidans*, fungi: *A. niger* and yeast: *Rhodotorula mucilaginosa*) for the extraction of Li from lepidolite (Li-containing mineral). They found that the heterotrophic fungal and yeast bioleaching was faster (40 days) than autotrophic bacterial consortium bioleaching (336 days). Altogether, the fungal bioleaching has the following advantages including fungal isolates having higher capacity to tolerate toxic components of spent LIBs, and the ability to grow in a broad range of pH (pH: 2–8, i.e., both acid and alkaline environment) with shorter lag phase (Bahaloo-Horeh et al., 2019).

**FIGURE 4:**
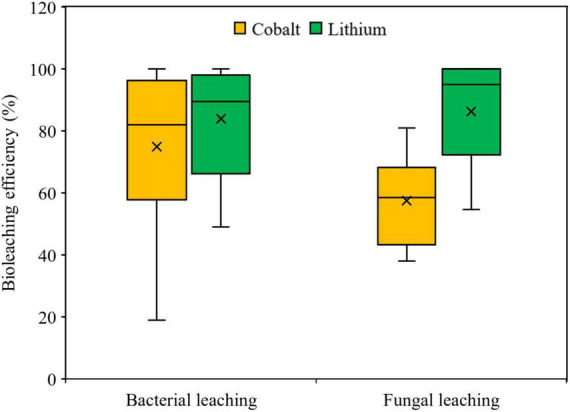
The Box and Whisker plot showing the comparative Co and Li leaching efficiency reported in literature using bacteria or fungi as the bioleaching agent.

For the comparative evaluation of variations in the dissolution of Co and Li in bacterial and fungal bioleaching, statistical analysis (Box and Whisker plot) was done using the literature data presented in Table 4 (bacterial leaching) and Table 5 (fungal leaching). The Box and Whisker plot (Figure 4) shows that in both types of bioleaching systems, Li dissolution was relatively higher than that of Co. Further analysis using the two-tailed Student *t*-test at 95% confidence interval (*P* ≤ 0.05) reveals that the difference between Co and Li dissolution efficiency was statistically significant (*P* < 0.05) for the fungal leaching, but not for the bacterial leaching. Between the two types of bioleaching systems, Co dissolution seems to be higher in the bacterial-based leaching system, whereas fungal leaching appears to promote more Li solubilization than the bacterial system (Supplementary Figure 4). However, more studies (specifically fungal leaching) are needed for better comparison of the metal leaching performance of the two types of biological methods.

### 5.5. Recovery of valuable metals from the bioleached medium

Multiple microbial-driven reactions in the bioleaching process facilitate the extraction of metals from the complex solid matrix of spent LIBs as well as convert the hazardous fractions of spent LIBs into non-hazardous form (Naseri et al., 2019b). Thus, the bioleached medium is usually highly rich in diverse valuable metals including Co and Li, but they are mainly in the dissolved form. From the circularity and economic perspectives, the dissolved secondary metals can be recycled/recovered by transferring the dissolved form of the metals into the solid form through microbial-driven ad/or chemical-driven precipitation reactions (Biswal et al., 2018). Biotechnological techniques such as bioprecipitation (e.g., employing metal reducing bacteria), biosorption (e.g., using living or dead biosorbents) and bio-electrochemical systems (e.g., microbial fuel cell and microbial electrolysis cell) can be applied for metal recovery (Sethurajan and Gaydardzhiev, 2021). The common precipitating agents used for the precipitation of metals include sulfides, carbonates and hydroxides. The pH of the medium plays a critical role for selective precipitation of the target metals. However, limited information is available on the recovery of valuable metals from the pregnant bioleached medium using biological methods. Biswal et al. (2018) used the fungal leaching solution for the recovery of Co and Li using the chemical precipitation method. Co recovery was attempted by adding three types of chemical reagents namely cobalt sulfide, cobalt hydroxide and cobalt oxalate which resulted in the Co recovery efficiency of 88–100%. Li was precipitated as lithium carbonate by adding sodium carbonate, and the Li recovery was 73.6%. Biogenic sulfide precipitation which is mediated by sulfate reducing bacteria (SRB) is used for metal recovery from secondary sources and natural ores (Sethurajan and Gaydardzhiev, 2021). The bioprecipitation reactions occur in the anoxic environment as shown in the following equations (Eqs. 6–8). Several studies have reported that SRB namely *Desulfovibrio vulgaris* are effective for bio-precipitation of Co as cobalt sulfide (CoS ⋅ xH_2_O) from the aqueous medium in sulfidic environment (Blessing et al., 2001; Mansor et al., 2020). In SRB-based bio-precipitation reactions (Supplementary Figure 5), organic carbon acts as an electron donor, while sulfate is acts as an electron acceptor (Sethurajan and Gaydardzhiev, 2021). Using acetic acid (CH_3_COOH) as the model organic carbon source, the bio-precipitation reactions are presented in Eqs. 9, 10 (Kumar et al., 2015).


(6) $$O\,M\,\left(O\,r\,g\,a\,n\,i\,c\,m\,a\,t\,t\,e\,r\right)+S\,O_{4}^{2-}\overset{S\,R\,B}{⟶}H_{2}\,S+H\,C\,O_{3}^{-}$$

(7) $$H_{2}\,S\to H\,S^{-}+H^{+}$$

(8) $$M^{2+}+H\,S^{-}\to M\,S_{\left(s\right)}+H^{+}\,\left(W\,h\,e\,r\,e\,M^{2+}\,M\,e\,t\,a\,l\,c\,a\,t\,i\,o\,n\right)$$

(9) $$C\,H_{3}\,C\,O\,O\,H+2\,H_{2}\,O\to 2\,C\,O_{2}+8\,H^{+}+8\,e^{-}$$

(10) $$S\,O_{4}^{2-}+8\,H^{+}+8\,e^{-}\to S^{2-}+4\,H_{2}\,O\to H_{2}\,S+M^{2+}$$

$$\to M\,S+2\,H^{+}$$

### 5.6. Bioleaching kinetics

Bioleaching kinetic studies help to understand the nature and mechanism of the leaching process (Baniasadi et al., 2019). However, limited information is currently available on the kinetics of spent LIBs bioleaching. Although kinetics of bioleaching process is usually slower than other recycling processes namely conventional hydrometallurgy and pyrometallurgy, a few studies have reported that while using metallic ions (e.g., Cu^2+^ and Ag^+^) as catalysts, the dissolution rate of metals was considerably enhanced (Zeng et al., 2013; Niu et al., 2015). Addition of catalytic ions accelerates electron transfer in the leaching solution (Zeng et al., 2013). With the application of 0.8 g/L Cu^2+^ into the bioleaching medium containing microbial consortia of *L. ferriphilum* (IOB) and *A. thiooxidans* (SOB), the solubilization rate of Zn and Mn from Zn–Mn batteries increased from 47.7 to 62.5% and from 30.9 to 62.4%, respectively (Niu et al., 2015). Additionally, the kinetic data were fitted with four different types of models (chemical reaction controlled model, shrinking sphere model, diffusion controlled model and product layer diffusion model), and the chemical reaction controlled model was most suitable to describe the kinetics data with the *R*^2^ value of 0.9783. However, another study from the same research group on spent LIBs bioleaching using microbial consortia of *Sulfobacillus* spp. (IOB) and *Alicyclobacillus* spp. (SOB) achieved 72% Co and 89% Li at pulp density of 2% (w/v) (Niu et al., 2014). However, the researchers reported that the product layer diffusion model was best fitted to describe the kinetic data (*R*^2^: 9,731). According to Baniasadi et al. (2019) the rate of bioleaching is controlled by the diffusion controlled model which is described as the shrinking core model. Sedlakova-Kadukova et al. (2020) also used the shrinking core model to describe the Li dissolution kinetics from lepidolite under three different microbial agent (bacteria, fungi and yeast) bioleaching systems. Other bioleaching studies on metal recovery from e-waste (e.g., printed circuit boards) also found that the shrinking core model was the most suitable for description of metal dissolution kinetics (Faraji et al., 2018; Arslan, 2021; Ilyas et al., 2022). In addition to metal ion catalysts, the application of ultrasonication (Nazerian et al., 2023) and reducing agents (Ghassa et al., 2020) is explored to enhance metal dissolution kinetics, but in-depth studies are needed to understand the associated kinetics mechanisms.

## 6. Factors impacting the performance of bioleaching process

The metal dissolution rate from spent LIBs in the bioleaching process depends on both biotic and abiotic factors (Moazzam et al., 2021; Sethurajan and Gaydardzhiev, 2021). The biotic factors include the type of microbial agents (bacteria vs. fungi). However, the abiotic factors include the leaching solution chemistry (e.g., concentration of nutrients and energy/carbon source and pH), environmental parameters (e.g., temperature), and other factors namely pulp density, spent LIBs particle size, aeration, and catalyst (Supplementary Table 3). The influence of the key parameters on the bioleaching performance is discussed below.

### 6.1. Composition of leaching medium

The quality and quantity of leaching medium components including nutrients and energy and carbon source considerably impact on the microbial growth and production of metabolites, and finally the bioleaching performance (Roy et al., 2021a). In bacterial leaching involving autotrophic microorganisms, various inorganic reagents such as S^0^, Fe^2+^ (e.g., FeSO_4_.7H_2_O) and pyrite (FeS_2_) are used as an energy source (Bahaloo-Horeh et al., 2019). To reduce the overall recycling cost, iron-containing waste materials like iron scrap are applied instead of commercial reagents (FeSO_4_.7H_2_O) for the bacterial leaching of spent LIBs and a similar level of metal recovery (Co and Ni) was achieved in both cases (Ghassa et al., 2020). In fungal leaching, which is mediated by the heterotrophic microbial agents, organic carbon (sucrose or glucose) is used as the carbon source (Roy et al., 2021a). Organic carbon containing industrial wastes namely vanasse can be utilized as carbon source in fungal leaching (Alavi et al., 2021).

In a recent study, with the increase of Fe_2_SO_4_ concentration from 45 to 150 g/L, the dissolution of Co was enhanced from 44.51 to 94.02%, while Li was increased from 42.92 to 60.30% (Roy et al., 2021b). Li et al. (2013) conducted the bioleaching study of spent LIBs using *A. ferrooxidans* at the Fe^2+^ concentration in the range between 25 and 65 mg/L, and an optimum Co recovery (48.2%) was achieved at the Fe^2+^ dose of 45 mg/L. The increase of Co recovery was related to the increase of the redox potential with the change of the Fe^2+^ dose in the leaching system. A study investigated the bioacids production by *A. niger* using sucrose as the carbon source with concentration ranging from 50 to 150 g/L (Bahaloo-Horeh and Mousavi, 2017). Higher production of various bioacids (citric acid: 26,478 mg/L, malic acid: 1,832.53 mg/L, gluconic acid: 8,433.76 mg/L) was obtained under the optimal sucrose dose of 116.90 g/L, fungal inoculum concentration of 3.45% (v/v) and leaching medium pH of 5.44.

In addition to carbon [e.g., organic carbon for heterotrophic and inorganic carbon (CO_2_) for autotrophic]/energy source, microorganisms require nutrients like N and P for their growth (cell synthesis) and activity (Mills et al., 2008; Biswal and Chang, 2022). Thus, inorganic reagents such as ammonium sulfate [(NH_4_)_2_SO_4_] (e.g., as a source of N) and potassium dihydrogen phosphate (KH_2_PO_4_)/dipotassium hydrogen phosphate (K_2_HPO_4_) (e.g., as a source of P) are added to the medium as nutrients to support the microbial growth (Marcinčáková et al., 2016). A study compared the (LiCoO_2_ content: 27.2%) bioleaching performance of a microbial consortia (*A. ferrooxidans* and *A. thiooxidans*) for spent LIBs in two different media: (1) synthetic nutrient medium (called 9K medium) containing all the nutrients and energy source, and (2) low nutrient medium containing only H_2_SO_4_ and S_0_ as the energy source (Marcinčáková et al., 2016). Nutrient-rich medium exhibited higher metal removal (Co: 67% and Li: 80%) than nutrients limiting medium (Co: 1.5% and Li: 35%). Overall, the bioleaching kinetics could be impacted by changes of the substrate concentration which serves as an electron donor and/or source of energy. Thus, bioleaching medium should be provided with an optimum concentration of substrates to achieve the highest microbial growth and metabolism as well as the highest recovery of metals from spent LIBs. The increase of substrate dose beyond the optimum concentration could show inhibitory effects to microbial activity and the optimum substrate doses could vary for different microbes.

### 6.2. Leaching medium pH

The acidity (pH) of the leaching medium generally controls the growth of leaching bacteria and bacterial-based catalytic reactions which is optimum up to nearly pH 3.5 (Mishra et al., 2008). Most of the acidophilic bacteria (e.g., IOB and SOB) show optimum growth at the pH range of 2.0–2.5 (Bosecker, 1997). Li et al. (2013) performed the bioleaching of spent LIB using *A. ferrooxidans* at the pH range of 1.0–4.0 and the highest Co recovery was obtained (47.6%) at the pH of 1.5. The fungal-based bioleaching can be performed in a wider pH range between 3.0–7.0 (Moazzam et al., 2021). Ijadi Bajestani et al. (2014) reported that *A. ferrooxidans*-based bioleaching at an initial pH of 1.0 with LIBs particle size of 1.62 μm and the initial Fe^3+^ concentration of 9.7 g/L demonstrated optimum removal of various metals (93.7% Co, 87% Ni and 67% Cd) from spent batteries (Ni-Cd and Ni-MH). The optimum pH for the *A. niger*-based bioleaching is nearly 5.0. In a fungal bioleaching test, it was observed that an initial pH of 5.44 with sucrose concentration of 116.90 g/L and inoculum size of 3.45% (v/v) results in a maximum production of various metabolites (citric acid, malic acid and gluconic acid) (Bahaloo-Horeh and Mousavi, 2017). In the bioleaching tests, the pH of the leaching medium usually increases (consumption of bioacids) initially after the addition of spent LIBs powder due to its alkaline nature of Li-based compounds in LIB (Heydarian et al., 2018). Li is an alkaline metal which highly reacts with water and produces lithium hydroxide in aqueous medium (Heydarian et al., 2018). Furthermore, in bacterial leaching, the oxidation of Fe^2+^ to Fe^3+^ by IOB resulted in a decrease of pH due to proton consumption as shown in the following equation (Eq. 11) (Ijadi Bajestani et al., 2014). Together, the leaching kinetics could be influenced by the changes of solution pH since it impacts the microbial growth and its activity. The optimum pH for a bioleaching process depends on the selected microbial agents and the operating systems.


(11) $$F\,e^{2+}+\frac{1}{4}\,O_{2}+H^{+}\overset{I\,O\,B}{⟶}F\,e^{3+}+\frac{1}{2}\,H_{2}\,O$$

### 6.3. Pulp density

The toxicity level of the leaching environment could change with the change of pulp density dose since metals and other hazardous components of spent LIB could exert toxicity effects to the microbial agents (Biswal et al., 2022). At higher pulp density, the metal ions (e.g., Co^2+^ and Li^+^) in the spent LIBs induce oxidative stress on the leaching microorganisms (Liu et al., 2020). In a simulated bioleaching experiments using acidophilic microbial consortium (*L. ferriphilum* and *S. thermosulfidooxidans*), at a pulp density of 4% (w/v) LiCoO_2_ powder, the intracellular ROS level in the mixed culture was enhanced from 0.82 to 6.02 in 24 h, which was nearly three times greater than the control test at 0% pulp density (2.04) (Liu et al., 2020). Niu et al. (2014) compared the valuable metals leaching efficiency at pulp densities of 1–4%, and observed that with the increase of pulp density from 1 to 4%, the amount of Co (declined from 52 to 10%) and Li (declined from 80 to 37%) dissolution was considerably reduced. The pulp density dose of 2% shows an optimum performance with 72% Co and 89% Li extraction being achieved. Naseri et al. (2019b) explored a two-step bioleaching of various metals from the spent lithium-ion coin cell at various pulp densities (10–50 g/) using the *A. thiooxidans*, and observed that the metal extraction decreased at higher pulp densities. The pulp density of 30 g/L resulted in optimum metal dissolution with 60% Co, 99% Li and 20% Mn removal. The decrease of metal removal efficiency at the higher pulp density is due to the reduction of microbial growth by environmental toxicity, the increase of viscosity of leaching solution and the reduction of oxygen transfer (Naseri et al., 2019b). In total, an appropriate pulp density should be provided to the bioleaching system to achieve maximum microbial growth and metal extraction. With the increase of pulp density, the oxygen mass transfer may decrease due to the increase of viscosity of the leaching medium which ultimately could reduce the metal extraction kinetics (Roy et al., 2021a). For the commercialization, bioleaching at higher pulp density is required.

### 6.4. Temperature

Temperature strongly influences the microbial growth, and hence impacts the bioleaching efficiency (Niu et al., 2014). A majority of SOB and IOB as well as fungal species can grow well between 28 and 30°C (Bosecker, 1997). Using a mixed culture of *Alicyclobacillus* spp. (SOB) and *Sulfobacillus* spp. (IOB), Niu et al. (2014) investigated the effects of various temperatures (30–40°C) on metal bioleaching from spent LIBs with a mixed bacterial culture, and found that with the increase of temperature from 30 to 35°C, the leaching of Co and Li increased from 52 and 78% to 72 and 89%, respectively. Further increase of temperature to 40°C resulted in the decrease of leaching efficiency which is possible due to inhibition of microbial growth (Niu et al., 2014). The bioleaching experiments are usually conducted in the temperature range between 22–35°C (Moazzam et al., 2021). Together, temperature is considered as one of the critical factors which impacts the leaching kinetics. The leaching kinetic increases up to the optimum temperature, and then decreases due to reduction of microbial growth and its activity. Additionally, the changes of temperature impact the thermodynamics (e.g., Gibb’s free energy) of various biochemical reactions according to the Arrhenius law of thermodynamics (Niu et al., 2014).

### 6.5. Aerobic environment (dissolved oxygen level)

Most of the acidophilic microorganisms, both bacteria (e.g., *A. ferrooxidans*) and fungi (e.g., *A. niger*) grow well under aerobic environments (Bosecker, 1997; Putra et al., 2022; Nazerian et al., 2023). Hence, sufficient oxygen/air should be supplied to the leaching medium (e.g., through aeration, stirring or shaking) to obtain optimum microbial activities and metal leaching efficiency. *A. ferrooxidans* gets energy for the growth through the oxidation of substrate, ferrous ions (electron donor) (i.e., oxidation of Fe^2+^ to Fe^3+^) in which the dissolved oxygen (O_2_) acts as a terminal electron acceptor (Liang et al., 2016; Liu et al., 2020). A recent bioleaching study using *A. ferrooxidans* isolated from the acid mine drainage reported that Co extraction from spent LIBs was nearly 74% with aeration (stirring) of the leaching medium, while Co leaching was reduced to nearly 52% without aeration (Putra et al., 2022). Overall, in aerobic bioleaching system, O_2_ acts as an electron acceptor. Thus, sufficient dissolved oxygen should be available in the leaching medium to achieve faster leaching kinetics.

### 6.6. Addition of catalysts

Bioleaching is usually considered as the slow kinetics process due to inhibition of growth and metabolism of leaching microorganism by toxic effects of high concentration metals (Zhang et al., 2023). Hence, to accelerate the metal dissolution kinetics, several metallic ions (e.g., Ag^+^, Cu^2+^, Bi^3+^, Hg^2+^, and Co^2+^) are added which accelerate the electron transfer and improve the metal removal performance (Niu et al., 2015). With the addition of 0.75 g/L of copper ions (Cu^2+^), the bioleaching of Co from spent LIBs remarkably increased from 43.1 to 99.1% in 10 days leaching period (Zeng et al., 2012). Additional bioleaching experiments from the same research group reported that with the supplementation of leaching medium with 0.02 g/L of silver ions (Ag^+^), the Co leaching rate was almost doubled within 7 days, i.e., the amount of Co dissolution increased from 43.1 to 98.4% (Zeng et al., 2013). Noruzi et al. (2022) also found similar results of the enhancement of metals extraction efficiency from spent LIBs with the addition of silver ions (0.02 g/L), i.e., up to 99.95% Co and Ni leaching was observed by the supplementation of leaching (two-step approach) medium with silver ions. The copper ion (Cu^2+^)-based (Eqs. 12, 13) and silver ion (Ag^+^)-based (Eqs. 14–16) catalytic reactions for the removal of Co and Li from spent LIBs are presented in Roy et al. (2021a) and Golmohammadzadeh et al. (2022).


(12) $$C\,u^{2+}+2\,L\,i\,C\,o\,O_{2}↔C\,u\,C\,o_{2}\,O_{4}+2\,L\,i^{+}$$

(13) $$C\,u\,C\,o_{2}\,O_{4}+6\,F\,e^{3+}↔6\,F\,e^{2+}+C\,u^{2+}+C\,o^{2+}+2\,O_{2}$$

(14) $$A\,g^{+}+L\,i\,C\,o\,O_{2}↔A\,g\,C\,o\,O_{2}+L\,i^{+}$$

(15) $$A\,g\,C\,o\,O_{2}+3\,F\,e^{3+}↔3\,F\,e^{2+}+A\,g^{+}+C\,o^{2+}+O_{2}$$

(16) 4⁢F⁢e2++O2+ 4⁢H+⟶B⁢a⁢c⁢t⁢e⁢r⁢i⁢a 4⁢F⁢e3++ 2⁢H2⁢O

In addition to metallic catalyst-based bioleaching, a few studies have applied ultrasonication (called sonobioleaching) to accelerate the leaching efficiency (Nazerian et al., 2023). Without ultrasonication, the leaching of Co, Li, Mn and Ni was 13, 57, 42 and 25%, respectively, with *A. ferrooxidans* at the pulp density of 10 g/L (Nazerian et al., 2023). However, with the application of ultrasonication (203.5 W for 0.5 h), the metal leaching efficiency was increased (Co: 19%, Li: 57%, Mn: 50% and Ni: 34%) and the leaching time was reduced to nearly half (shortened from 24 h to 12 h). The increase of bioleaching efficiency by the application of ultrasound is due to the following four mechanisms such as (1) increase of convective penetration in the leaching medium by disintegration of particles, (2) increase of temperature and pressure of the leaching medium by cavitation, (3) enhancement of the homogeneous and heterogeneous reactions (having metals as the catalysts/reactants) by ultrasound application, and (4) generation of various reactive radical species according to the following reactions (Eqs. 17–20), followed by the enhancement of the concentration of ferric ion (Eqs. 21–23) in the leaching medium that accelerates/stimulates the reaction rate (Nazerian et al., 2023). Overall, in the catalysts-based bioleaching system, an appropriate dose of catalysts should be applied to the leaching medium to obtain maximum microbial growth and leaching efficiency. The addition of catalysts lowers the activation energy, and thus accelerates the reaction rate (Bahaloo-Horeh et al., 2019).


(17) $$H_{2}\,O\to H^{.}+H\,O^{.}$$

(18) $$H^{.}+O_{2}\to H\,O\,O^{.}$$

(19) $$2\,H\,O^{.}\to H_{2}\,O_{2}$$

(20) $$2\,H\,O\,O^{.}\to H_{2}\,O_{2}+O_{2}$$

(21) $$F\,e^{2+}+O\,H^{.}\to F\,e^{3+}+O\,H^{-}$$

(22) $$F\,e^{2+}+H\,O_{2}^{.}\to F\,e^{3+}+H\,O_{2}^{-}$$

(23) $$F\,e^{2+}+H_{2}\,O_{2}\to F\,e^{3+}+O\,H^{-}+O\,H^{.}$$

### 6.7. Spent LIBs particle size

Mass transfer is one of the key factors which influences the bioleaching performance (Bahaloo-Horeh et al., 2019). The availability of contact surface of the particles of spent LIBs used for leaching impacts the mass transfer rate. Generally, with the decrease of particle size (i.e., higher surface are), the contact surface increases, and as a result the mass transfer also increases (Bahaloo-Horeh et al., 2019). The upsurge of mass transfer contributes to the higher removal of metals from spent LIBs. Appropriate mesh size is usually used to sieve and collect the desired smaller size particles spent LIBs powder after crushing and milling (Mishra et al., 2008). In a majority of the studies, the size of spent LIBs powder used in bioleaching tests largely varied between less than 75 and 300 μm (Mishra et al., 2008; Bahaloo-Horeh et al., 2018; Biswal et al., 2018). Nonetheless, a few studies used commercial LiCoO_2_ powder for the bioleaching tests and the particle size varied between 105 and 130 μm (Liu et al., 2020). Together, to achieve higher metal extraction in bioleaching, an appropriate LIB particle size should be used since the mass transfer is limited at bigger particle size. Additionally, the biofilm development and microbe-metal interactions may be higher in smaller LIB particles due to high surface area, and stronger microbe-metal interactions could result in faster leaching kinetics.

## 7. Insights into bioleaching mechanisms: metal dissolution by microbe-material interactions

**FIGURE 5:**
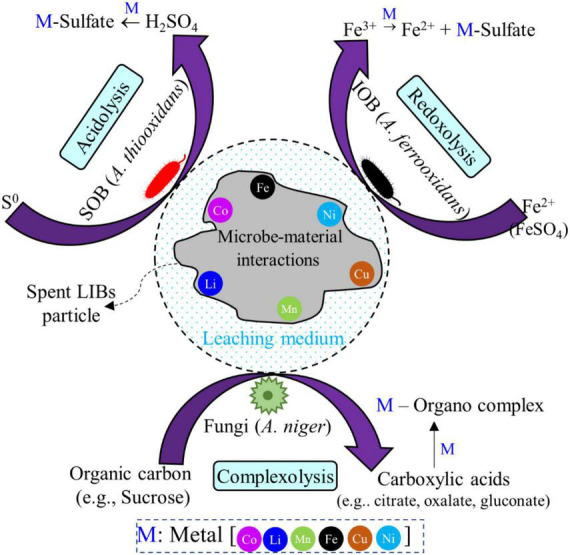
Key biochemical mechanisms for removal of metals from spent LIBs [adapted and modified from a previous study (Sethurajan and Gaydardzhiev, 2021)].

Several biochemical mechanisms are proposed to explain the bioleaching reactions (Işıldar et al., 2019). The bioleaching reactions are broadly categorized into three groups namely (1) acidolysis, (2) redoxolysis and (3) complexolysis (Figure 5) based on the types of energy and carbon sources used as well as the type of biological production of metabolites (e.g., bioacids) (Botelho Junior et al., 2021; Sethurajan and Gaydardzhiev, 2021). In acidolysis, metal transformation from insoluble to soluble form occurs by the produced bioacids and/or protons. In redoxolysis, the microbes are attached to the surface of waste materials to be leached through the biofilm formation and the extracellular polymers (EPS), triggering the metal solubilization due to electron transfer between the solid mineral in waste materials and the microbes. In the case of complexolysis, the metabolites (bioacids) produced by the microbes form the soluble metal-organic complex through chelation and complexation reactions (Sethurajan and Gaydardzhiev, 2021). For the bacterial–based leaching, two types of leaching mechanisms are proposed: (1) direct (contact) leaching, and (2) indirect (non-contact leaching) (Bahaloo-Horeh et al., 2019; Roy et al., 2021a). The direct mechanism mainly occurs in one-step and two-step bioleaching tests where there is a physical contact between the microorganisms and the spent LIBs particles (Bahaloo-Horeh et al., 2019). However, the indirect mechanisms are mainly applicable to the cells free spent medium bioleaching test (Bahaloo-Horeh et al., 2019). The bioleaching mechanism for the removal of Li is different from that of other metals (Co, Mn, and Ni), For example, Li leaching is mainly driven by the non-contact mechanism (acidolysis), while the contact mechanism [acid solubilization plus reduction of insoluble form of metals (Co^3+^, Mn^4+^, and Ni^3+^) by Fe^2+^] contributes to the removal of Co, Mn and Ni from spent LIBs (Xin et al., 2016). The extracellular polymeric substances (EPS) secreted by the bacteria play an important role in metal dissolution since strong attachment occurs between bacterial cell and spent LIBs particle through EPS by hydrophobic and electrostatic forces (Wang et al., 2018). Moreover, EPS concentrate Fe^2+^/Fe^3+^ cycle inside the battery particle which accelerate the metal removal by reductive mechanism. EPS increases the electronic potential which accelerates electron transfer and metal solubilization. A few studies have reported that biosorption and bioaccumulation contribute to metal removal from spent batteries specifically in fungal leaching (Bahaloo-Horeh et al., 2019; Dusengemungu et al., 2021). Biosorption is a process of accumulation of metals onto the biomass through numerous physicochemical processes (e.g., adsorption). Bioaccumulation is a process of the transport of soluble metal ions into the living biomass through cell membrane which is facilitated by the different functional groups (amine, carboxyl, hydroxyl, phosphate and sulfate) present in the fungal mycelium (Dusengemungu et al., 2021).

### 7.1. Bacterial bioleaching mechanisms

#### 7.1.1. Direct leaching

Under the direct leaching, the microbes attachment to the surface of the spent LIBs particle facilitates the electron transfer reactions (or electrochemical interactions) between the metal substrate in spent LIBs and the reduced metal ions (usually added externally) (Roy et al., 2021a). Since spent LIBs hardly contain any iron-sulfur containing minerals, S^0^ and Fe^2+^ (e.g., in the form of FeSO_4_) are usually added externally as a source of energy and electron donor to promote bacterial leaching. The direct microbial interactions between bacteria (e.g., SOB) and spent LIBs results in the formation of biogenic inorganic acid, H_2_SO_4_ due to the oxidation of S^0^ and the concurrent oxidation of Fe^2+^ to Fe^3+^ also occurs (Eqs. 24–27) (Bahaloo-Horeh et al., 2019; Roy et al., 2021a). Both H_2_SO_4_ and Fe^2+^ act as oxidizing agents and facilitate the mobilization of metals from the spent LIB solid matrices. Xin et al. (2009) reported that acid-based solubilization (acidolysis) was the sole mechanism for valuable metal recovery from spent batteries in the sulfur (S^0^)-based bioleaching system, whereas a combined effects of acid solubilization (acidolysis) and Fe^2+^ facilitated reduction (redoxolysis) contributed for the metal removal in the FeS_2_ or S + FeS_2_ bioleaching system.


(24) $$S^{0}+H_{2}O+\frac{3}{2}O_{2}\overset{SOB\;(e.g.,\;A.\;thiooxidans)}{\to }H_{2}SO_{4}$$

(25) $$H_{2}SO_{4}+M\left(s\right)\to MSO_{4}\left(aq\right)+2H^{+}\left(M=MetalsinspentLIBs\right)$$

(26) $$4\,L\,i\,C\,o\,O_{2}+3\,H_{2}\,S\,O_{4}\to C\,o_{3}\,O_{4}\,\left(s\right)+2\,L\,i_{2}\,S\,O_{4}\,\left(a\,q\right)+C\,o\,S\,O_{4}\,\left(a\,q\right)$$

$$+3\,H_{2}\,O+\frac{1}{2}\,O_{2}$$

(27) 2⁢F⁢e2++12⁢O2+2⁢H+⟶B⁢a⁢c⁢t⁢e⁢r⁢i⁢a 2⁢F⁢e3++H2⁢O

#### 7.1.2. Indirect leaching

**FIGURE 6:**
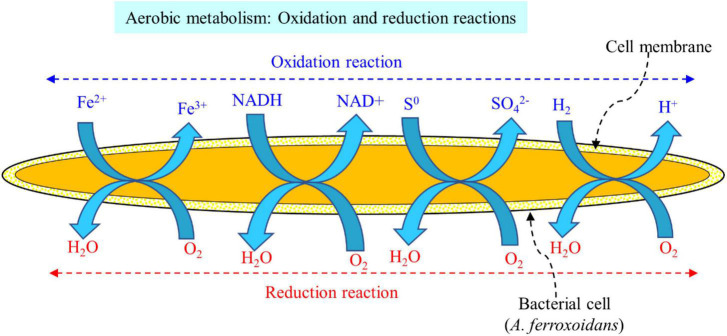
Oxidation and reduction reactions involving by aerobic metabolism of *Acidithiobacillus ferrooxidans* which contribute to the metal leaching [adapted and modified from a previous study (Valdés et al., 2008)].

The indirect leaching is carried out by the lixiviants produced by the bacteria which chemically oxidize the reduced metal substrates in the spent LIBs (Bosecker, 1997). The IOB oxidizes Fe^2+^ to Fe^3+^, then reduction reactions of Fe^3+^ lead to the production of protons (H^+^ ions) which enhances the metals recovery efficiency (Eqs. 28–31). The aerobic metabolism of *A. ferrooxidans* is presented in Figure 6.


(28) $$4Fe^{2+}+O_{2}+4H^{+}\overset{IOB\;(e.g.,\;A.\;ferrooxidans)}{\to }4Fe^{3+}+2H_{2}O$$

(29) $$F\,e^{3+}+H_{2}\,O\to F\,e\,{\left(O\,H\right)}^{+}+H^{+}$$

(30) $$F\,e^{3+}+2\,H_{2}\,O\to F\,e\,{\left(O\,H\right)}_{2}^{+}+2\,H^{+}$$

(31) $$F\,e^{3+}+3\,H_{2}\,O\to F\,e\,{\left(O\,H\right)}_{3}^{+}+3\,H^{+}$$

### 7.2. Fungal bioleaching mechanism

The fungal bioleaching mechanism mainly involves organic carboxylic acids (e.g., citric acid, oxalic acid and gluconic acid) produced by the heterotrophic fungal species during aerobic metabolism using sucrose as the carbon source (Bahaloo-Horeh and Mousavi, 2017). The metal solubilization by organic acids is mainly driven by the acidolysis and complexolysis reactions (by protons released by bioacids). However, the bioacids also change the oxidation potential of the leaching media, i.e., redoxolysis reaction (by anions from bioacids, e.g., citrate, oxalate, gluconate, etc.) and also contribute to the metal solubilization (Eqs. 32–43) (Biswal et al., 2018; Işıldar et al., 2019).


(32) $$C_{12}\,H_{22}\,O_{11}\,\left(S\,u\,c\,r\,o\,s\,e\right)+H_{2}\,O\to C_{6}\,H_{12}\,O_{6}\,\left(G\,l\,u\,c\,o\,s\,e\right)$$

$$+C_{6}\,H_{12}\,O_{6}\,\left(F\,r\,u\,c\,t\,o\,s\,e\right)$$

(33) $$C_{6}\,H_{12}\,O_{6}\,\left(G\,l\,u\,c\,o\,s\,e\right)+1.5\,O_{2}\to C_{6}\,H_{8}\,O_{7}\,\left(C\,i\,t\,r\,i\,c\,a\,c\,i\,d\right)+2\,H_{2}\,O$$

(34) $$C_{6}\,H_{8}\,O_{7}↔{\left(C_{6}\,H_{7}\,O_{7}\right)}^{-}+H^{+}$$

(35) $${\left(C_{6}\,H_{7}\,O_{7}\right)}^{-}+M^{+}\,\left(M\,e\,t\,a\,l\right)↔M\,\left[C_{6}\,H_{7}\,O_{7}\right]$$

(C⁢i⁢t⁢r⁢i⁢c⁢m⁢e⁢t⁢a⁢l⁢l⁢i⁢c⁢c⁢o⁢m⁢p⁢l⁢e⁢x)

(36) $$C_{6}\,H_{12}\,O_{6}\,\left(G\,l\,u\,c\,o\,s\,e\right)+4.5\,O_{2}\to 3\,C_{2}\,H_{2}\,O_{4}\,\left(O\,x\,a\,l\,i\,c\,a\,c\,i\,d\right)+3\,H_{2}\,O$$

(37) $$C_{2}\,H_{2}\,O_{4}↔{\left(C_{2}\,H\,O_{4}\right)}^{-}+H^{+}$$

(38) $${\left(C_{2}\,H\,O_{4}\right)}^{-}+M^{+}\,\left(M\,e\,t\,a\,l\right)↔M\,\left[C_{2}\,H\,O_{4}\right]$$

(O⁢x⁢a⁢l⁢i⁢c⁢m⁢e⁢t⁢a⁢l⁢l⁢i⁢c⁢c⁢o⁢m⁢p⁢l⁢e⁢x)

(39) $$C_{6}\,H_{12}\,O_{6}\,\left(G\,l\,u\,c\,o\,s\,e\right)+O_{2}\to C_{6}\,H_{10}\,O_{6}\,\left(G\,l\,u\,c\,o\,n\,o\,l\,a\,c\,t\,o\,n\,e\right)+H_{2}\,O_{2}$$

(40) $$C_{6}\,H_{10}\,O_{6}\,\left(G\,l\,u\,c\,o\,n\,o\,l\,a\,c\,t\,o\,n\,e\right)+H_{2}\,O\to C_{6}\,H_{12}\,O_{7}$$

$$\left(G\,l\,u\,c\,o\,n\,i\,c\,a\,c\,i\,d\right)+H_{2}\,O_{2}$$

(41) $$H_{2}\,O_{2}\to H_{2}\,O+\frac{1}{2}\,O_{2}$$

(42) $$C_{6}\,H_{12}\,O_{7}↔{\left(C_{6}\,H_{11}\,O_{7}\right)}^{-}+H^{+}$$

(43) $${\left(C_{6}\,H_{11}\,O_{7}\right)}^{-}+M^{+}\,\left(M\,e\,t\,a\,l\right)↔M\,\left[C_{6}\,H_{11}\,O_{7}\right]$$

(G⁢l⁢u⁢c⁢o⁢n⁢i⁢c⁢m⁢e⁢t⁢a⁢l⁢l⁢i⁢c⁢c⁢o⁢m⁢p⁢l⁢e⁢x)

## 8. Sustainability of bioleaching method for recycling of spent LIBs

Our literature review shows that most of previous studies mainly focused on the recycling of spent LIBs using various technologies, but limited information is available about the sustainability assessment of recycling technologies, especially the bioleaching method. The comparison of bioleaching and other recycling methods (e.g., pyrometallurgical and hydrometallurgical) from the sustainability viewpoint is usually done by performing comprehensive LCA which considers most of the environmental impacts of a recycling technique (Villares et al., 2016). A detailed LCA is required for scaling of the bioleaching process for industrial applications (Roy et al., 2022). A few studies have also performed techno-economic analysis (TEA) and energy assessment of the bioleaching process. According to a very recent study, the LCA results show the reduction of global warning potential (GWP) by nearly 8 times with the recycling of spent LIBs by bioleaching methods using *Gluconobacter oxydans* bacteria (6–19 kg CO_2_ equivalent GWP per kg of recovered Co) compared to other technologies (e.g., hydrometallurgy using HCl, 43–91 kg CO_2_ equivalent GWP per kg of recovered Co) (Alipanah et al., 2023). The TEA analysis projected a possible average profit of 21% for the processing of 10,000 tons/year of black mass (mostly cathode materials from spent LIBs). The economic viability of the bioleaching method is highly dependent on the purchasing price of spent LIBs (costs of collection and transportation). Moreover, the cost of chemical reagents used as the energy sources reagent (e.g., iron sulfate) and their consumption rate also have effects on the economic feasibility of the bioprocess (Alipanah et al., 2023). Sun et al. (2016) performed the LCA of recycling of spent Zn-Mn batteries using bacterial consortia (*Alicyclobacillus* spp. and *Sulfobacillus* spp.) at the pilot-scale operation mode. Among the tested 18 environmental impact parameters, the two parameters namely human toxicity (62.7 kg 1, 4- dichlorobenzene equivalent per kg of battery treatment) and marine ecotoxicity (0.46 kg 1, 4- dichlorobenzene equivalent per kg of battery treatment) were the main components of the environmental impact that get much attention. Among the various recycling processes, the pre-treatment processes such as mechanical cutting and crushing of spent LIBs accounted for the highest environmental impact.

Although limited information is available on the TEA for spent LIBs recycling using the bioleaching method, Işıldar (2018) compared the TEA for the recycling of printed circuit boards (PCBs) using three different routes (chemical, biological and hybrid approaches consisting of chemical plus biological methods). Notably, the total costs (a combination of operational costs and capital investment costs) for PCBs recycling using the biological method (EUR 0.616/kg PCB) was lower than that of the chemical (EUR 0.67/kg PCB) and hybrid methods (EUR 1.008/kg PCB). Thus, the TEA results suggest that the biological process is the most economically feasible method for the recovery of metals from e-wastes. Nevertheless, other critical factors including the potential environmental impacts, recovery yield and possible revenue generation need to be considered for selection of a specific recycling technology (Moazzam et al., 2021). For the overall costs associated with the recycling of spent LIBs, the cost of purchasing of spent LIBs was the major contributor (62–89%) of the total recycling cost (Alipanah et al., 2023). Boxall et al. (2018) computed the economic value of various metals recovered from the spent LIB by employing a sequential batch leaching process with biogenic ferric iron (mixed culture of IOB and SOB) and 100 mM H_2_SO_4_, and they reported the requirements to achieve the potential economic value of US$10,769 (Co: $9,558, Cu: $602, Ni:$332, Li: $257 and Mn:$20) for processing of one ton of spent LIBs. The maturity level which is measured by the technology readiness levels (TRL) of the biological method for e-wastes recycling seems to be lower [TRL > 4 (exploratory stage), operation at the column and tank reactors] compared to pyrometallurgical and hydrometallurgical methods (TRL > 6) (Moazzam et al., 2021).

## 9. Future research directions

• In most of the existing bioleaching studies, specific groups of microbial agents (e.g., *A. thiooxidans* and *A. ferrooxidans* in bacterial leaching, and *A. niger* in fungal leaching) are studied for their bioleaching performance. However, efforts should be made in future for isolation of acidophilic microbes from the acidic and metal contaminated sites (e.g., acid-mine drainage), followed by assessment of their bioleaching capacity.
• Since spent LIBs contain diverse toxic elements including critical metals and organic electrolytes/solvents, synthetic biology-based techniques (e.g., genetic engineering) can be applied to modify the metal tolerance genes in the microbial genome to enhance its tolerance level to the toxic elements of spent LIBs. The overall recycling cost may decease using the genetically modified microbes (engineered microbes) since a few pre-treatment processes (e.g., washing and drying of battery powder) can be omitted.
• Although numerous research works are performed on critical metal dissolution using bacterial and fungal bioleaching, limited information is currently available about the recovery of highly concentrated dissolved metal ions from the pregnant bioleached solution using biological methods, e.g., bio-precipitation employing the metal reducing bacteria. Recovery of high-grade valuable metals from spent LIBs electrodes would contribute not only to the economy, but also to achieving the circularity and a closed-loop bioprocess.
• Future studies should provide a better understanding of the leaching kinetics and thermodynamics of the bioleaching process. This would in turn help to gain insights into the potential mechanisms involved in the microbial-mediated metal solubilization.
• The existing literature has largely focused on the optimization of operating parameters to enhance the critical metal dissolution efficiency from spent LIBs powder, but more attention should be given to assess the economic, energy and environmental sustainability assessment of the bioleaching method.

## 10. Discussion and conclusion

Recycling of spent LIBs is necessary from the perspectives of sustainability, circular economy and environmental protection (Zeng et al., 2014; Mao et al., 2022). This review presents a comprehensive analysis of the current developments on the recovery of valuable metals (mainly Co and Li) from spent LIBs using microbial agents namely bacterial and fungal species. The efficiency of bioleaching processes reported in literature has large variations which could be due to differences in experimental, operational and/or environmental conditions adopted in different works including the type of microbial agents employed, changes of leaching medium chemistry, changes of spent LIBs chemistry (cathode materials), and environmental conditions (temperature) (Moazzam et al., 2021; Sethurajan and Gaydardzhiev, 2021). Between the two types of microbial agents, fungal leaching seems to result in higher overall valuable metal solubilization yield than bacterial leaching because heterophilic fungi exhibit high level of tolerance to the toxic leaching environment. Additionally, it produces multiple metabolites (organic carboxylic acids) than bacteria (Biswal et al., 2018, 2022). Fungi adopt different pathways to maintain their activity in toxic environments, for example, transformation of the solubilized form of metals into their insoluble forms by reaction of the produced bioacids (e.g., precipitation of metal oxalate).

Additionally, biosorption and intracellular bioaccumulation hinder the transport of metal ions into cells. The metal solubilization capacity of microbial agents can be improved by the adaptation method (i.e., enhancement of microbial resistance to toxic metals) by exposing the microbes to the toxic environment initially by gradually increasing spent LIBs pulp density (Bahaloo-Horeh et al., 2018), adding synthetic lithium and cobalt salt solutions (Lobos et al., 2021) or by adding metallic catalysts (Ag^+^ or Cu^2+^ ions) (Zeng et al., 2012, 2013). A few studies have applied microbial consortia (e.g., mixed culture of IOB and SOB) in the bioleaching process (Heydarian et al., 2018; Ghassa et al., 2020), and obtained a high metal leaching yield. The synergetic interactions of microbial consortia with battery powder could facilitate the production of bioacids/oxidizing agents that in turn accelerate metal dissolution. Among the two types of valuable metals, the solubilization rate of Li seems to be higher than Co in both bacterial and fungal leaching systems which could be related to its unique physicochemical properties including high instability, high chemical activity as well strong hydration power (i.e., formation LiOH) in aqueous medium (Wang et al., 2012; Wei et al., 2021). Bioleaching is successfully applied for the extraction of valuable metallic resources from ores in mining industry (Zhang et al., 2023). However, bioleaching of metals from spent LIBs has largely been carried out in the laboratory-scale. At present, the TRL (technology readiness level) of bioleaching processes for the recycling of e-waste is nearly 4 (i.e., exploratory research) (Moazzam et al., 2021). Hence, more studies (mainly pilot-scale) are needed to better understand the changes of the bioleaching performance with the changes of various operating parameters as well as the associated mechanisms so that the scale-up of the bioleaching process can be carried out for industrial-scale applications.

The commercial application of the bioleaching method for critical metal recovery from spent LIBs is limited due to its slow kinetics (Mishra et al., 2008). However, several techniques including application of metal catalyst (Cu^2+^ and Ag^+^) (Zeng et al., 2012, 2013), ultrasound treatment (called sonobioleaching) (Nazerian et al., 2023) are adopted to enhance the bioleaching kinetics and/or metal recovery. A few studies have reported that the chemical-biological hybrid systems were effective for optimum recovery of valuable metals from spent LIBs (Dolker and Pant, 2019) as the *Lysinibacillus*–citric acid hybrid system was very efficient specifically for Co recover (98%). The bioleaching method is sustainable than other recycling technologies (e.g., pyrometallurgical and hydrometallurgical) because LCA-based studies have reported that GWP of bioleaching is considerably lower than hydrometallurgy (Alipanah et al., 2023). TEA also shows that bioleaching is economically feasible than other recycling methods (e.g., chemical and hybrid technologies) (Işıldar, 2018).

The synthesis of LIBs (specifically electrode materials) largely depends on the natural resources which are limited, for example, natural graphite for anode and critical metals like Co and Li for cathode synthesis (Olivetti et al., 2017). Biomass or bio-waste which are usually rich in organic carbon and considered as a source of renewable energy (Ahmed et al., 2022). Presently, great interest is given for the development of biowaste-based electrode materials to produce eco-friendly LIBs (Ahmed et al., 2022; Ho et al., 2022). The electrical stability of biowaste-based LIBs is comparable to LIBs synthesized using natural materials.

To achieve energy sustainability, at present significant interest is given worldwide on the development of renewable energy (green energy). The potential sources of renewable energy include biomass energy, solar energy, hydro energy, wind energy, tidal energy and geothermal energy (Kalyani et al., 2015). The key advantages of renewable energy include they are abundant, renewable and environmentally friendly due to low or zero greenhouse gas (e.g., CO_2_) emission (Kalyani et al., 2015). For biomass-based energy, various types of biomass or biowastes are used as a feedstock material for conversion them into bioenergy using biotechnological-based methods (Anwar et al., 2014). For example. lignocellulosic waste (e.g., agricultural waste) are used for the production of bioethanol (Anwar et al., 2014), whereas microalgae biomass is used for production biodiesel (Ray et al., 2022). Anaerobic digestion is also a promising technology for conversion of biomass to bioenergy (e.g., biomethane) (Milledge et al., 2019). Genetic and metabolic engineering tools are applied to engineer the organisms to enhance the bioenergy production (Brar et al., 2021).

The major conclusions drawn from this review on the recovery of valuable metals from spent LIBs using microbial agents are presented here.

• Spent LIBs are usually rich in various valuable metals namely Co and Li, but their concentrations vary with the change of cathode material chemistry.
• Lithium and cobalt-based LIBs are widely used in electrical and electronic devices due to their high energy density.
• Bioleaching is an eco-friendly and green technology which looks promising for effective recovery of valuable metals from spent LIBs.
• Acidophilic microorganisms including chemolithotrophic bacteria (IOB and SOB) and heterotrophic filamentous fungi (e.g., *A. niger*) are widely used for the dissolution of valuable metals from spent LIBs.
• Bioacids produced by the microbial agents [H_2_SO_4_ by bacteria and diverse organic carboxylic acids (e.g., oxalic, citric and gluconic acids) by fungi] mainly contribute to the metal dissolution.
• The major mechanisms involved in the solubilization of metals include acidolysis, redoxolysis and complexolysis.
• Several biotic (type of microbial agents) and abiotic factors (leaching medium composition, pH, pulp density, aeration, particle size of LIBs powder, temperature, etc.) considerably impact the critical metal recovery efficiency in bioleaching processes.
• The bioleaching process is thermodynamically feasible, and the process is also sustainable due to its minimal negative environmental impacts and cost-effective than other recycling technologies.
• In view of the promising resource recovery applications of the bioleaching process, efforts are needed to improve the technical maturity of this process toward its large-scale practical applications based on pilot studies and techno-economic assessments through multi-disciplinary collaboration.

## Author contributions

BB: conceptualization, investigation, methodology, writing—original draft, and review and editing. RB: conceptualization, supervision, funding, and writing—review and editing. Both authors contributed to the article and approved the submitted version.
