# Supplementary material for: Recovery of valuable metals from spent lithium-ion batteries using microbial agents for bioleaching: a review
Source: Front Microbiol. 2023 May 31;14:1197081. doi: 10.3389/fmicb.2023.1197081 (PMC10264615; doi:10.3389/fmicb.2023.1197081)
Supplement: Supplementary file 1 [file Data_Sheet_1.docx]

Supplementary Material

Recovery of Valuable Metals from Spent Lithium-ion Batteries using Microbial Agents for Bioleaching: A Review

Basanta Kumar Biswal ^1,^ * and Rajasekhar Balasubramanian ^1,^ *

^1^ Department of Civil and Environmental Engineering, National University of Singapore, Singapore 117576, Singapore

*** Correspondence:** pupun.biswal@gmail.com (BK Biswal); ceerbala@nus.edu.sg (R. Balasubramanian)

# Supplementary Figures and Tables

## Supplementary Figures


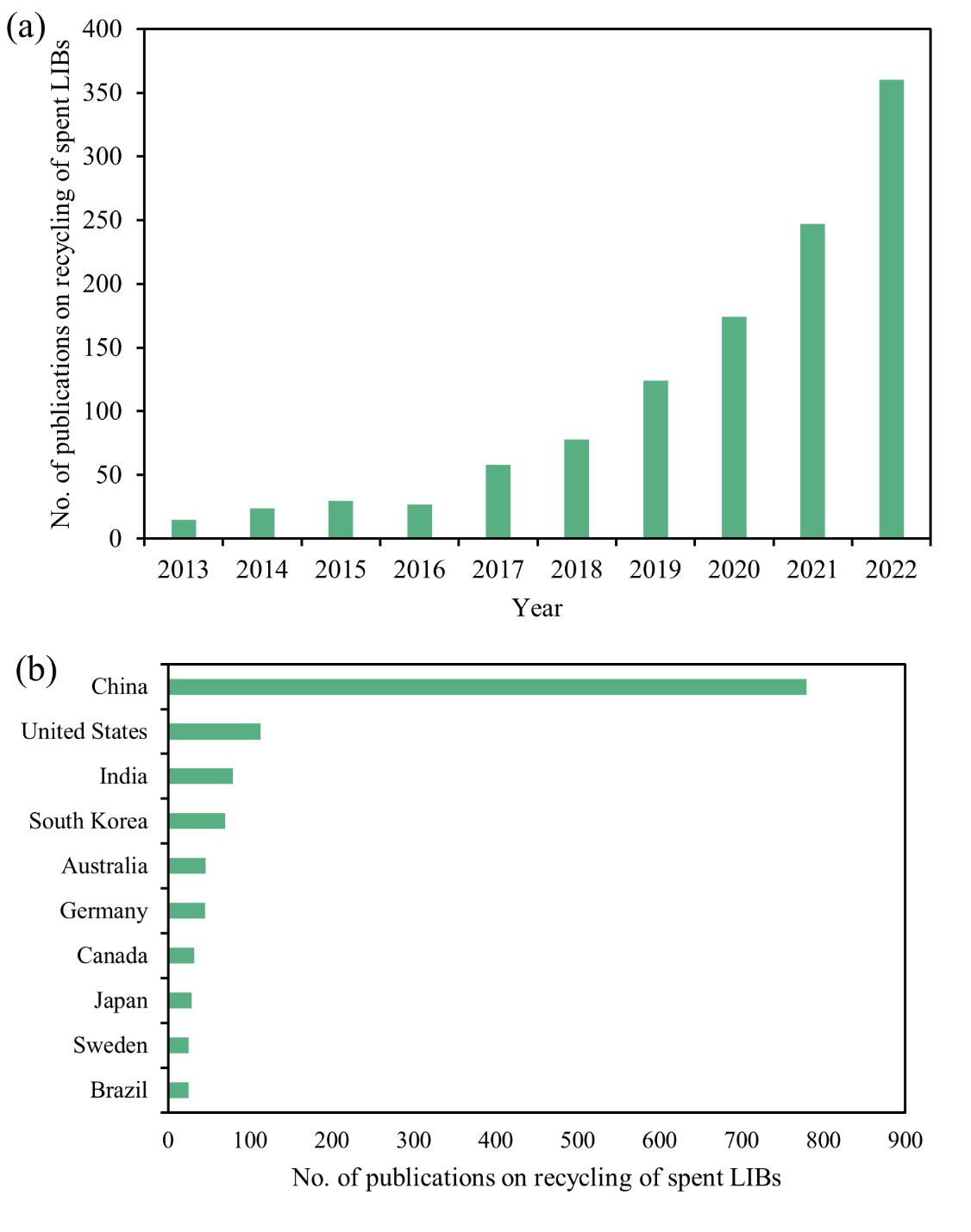


**Supplementary Figure 1.** Publication trend on the recycling of spent Lithium-ion batteries in last ten years (2013 – 2022) and the distribution of publications in various countries.


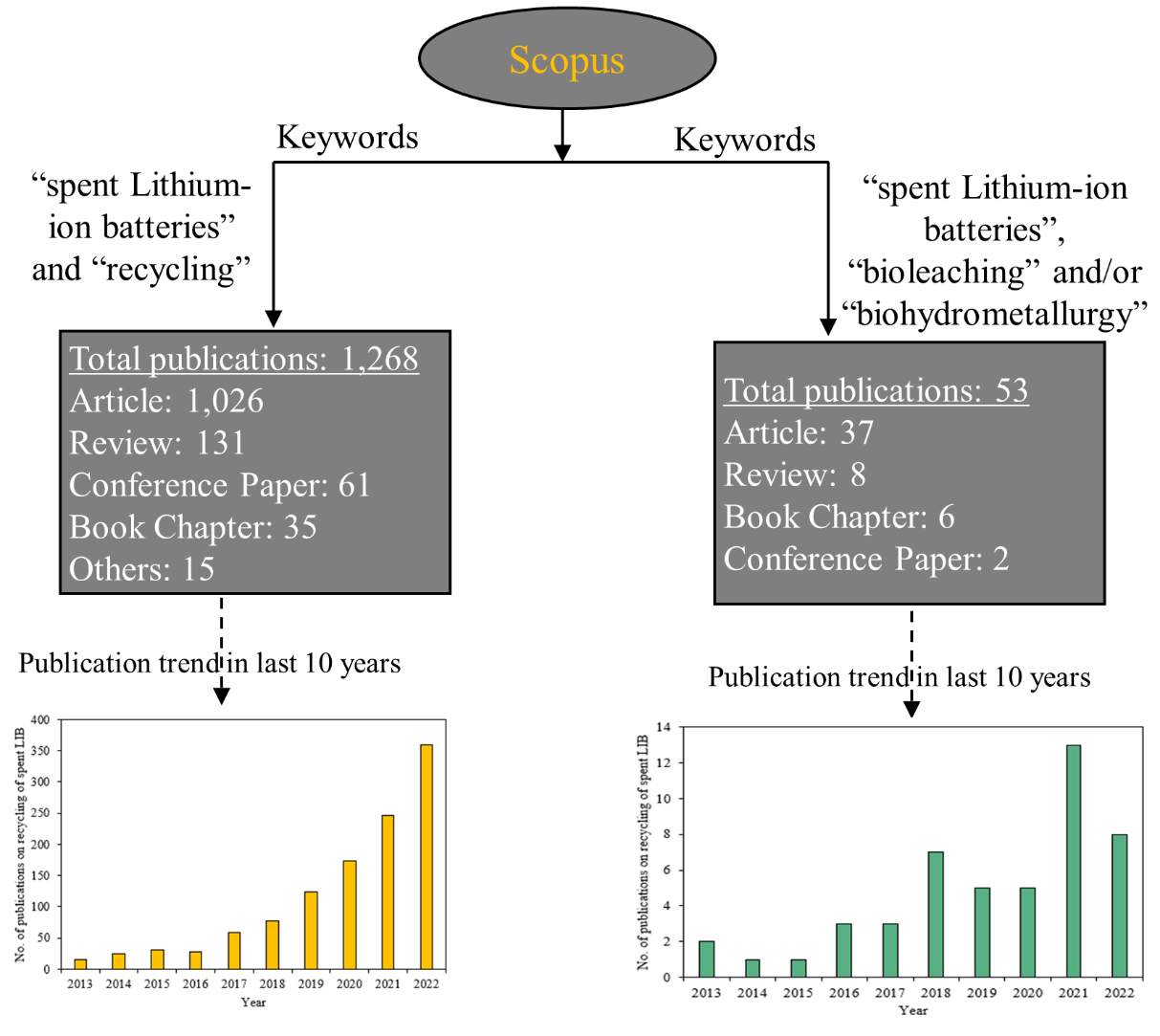


**Supplementary Figure 2.** Flowchart for the literature review method.


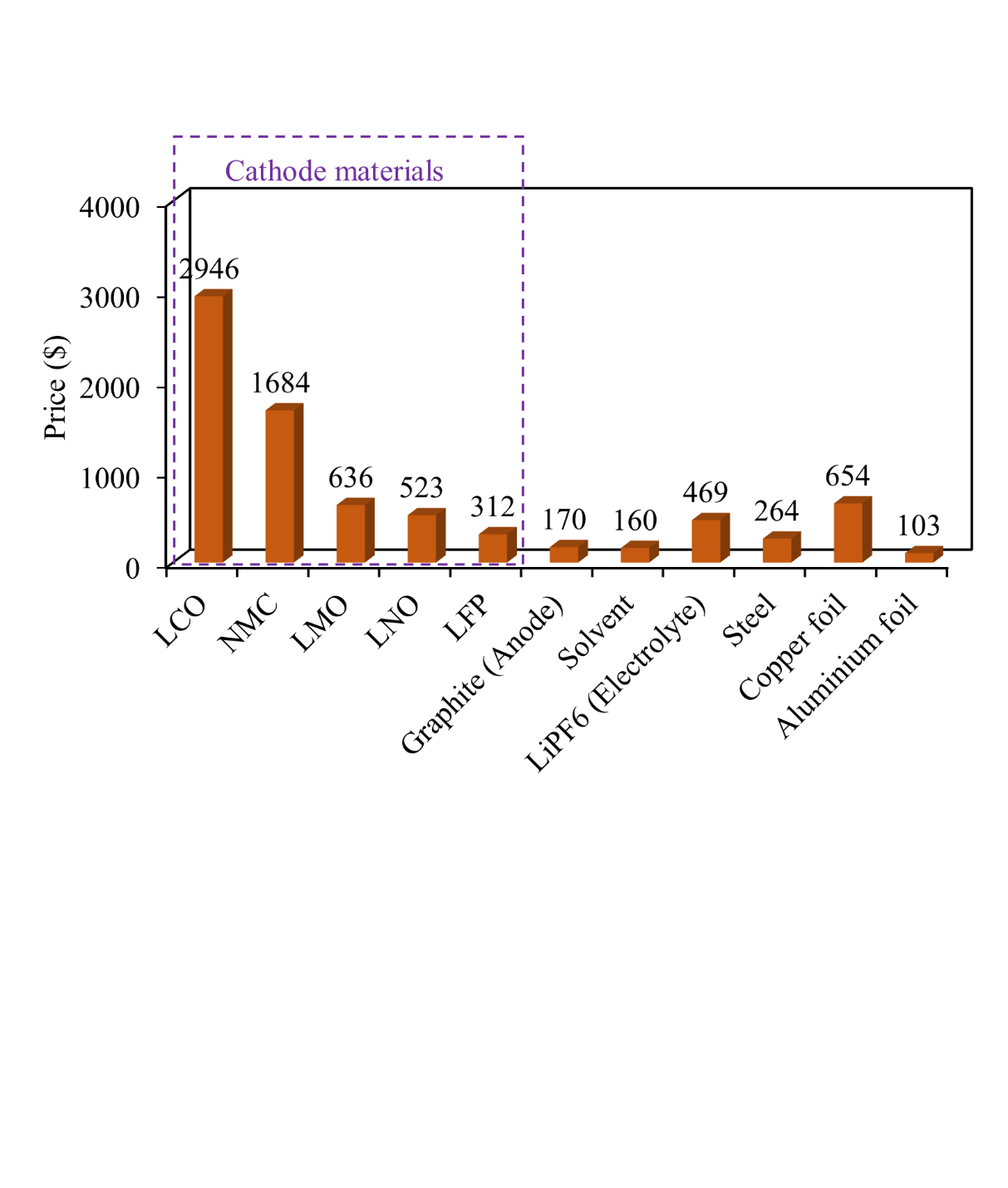


**Supplementary Figure 3.** The cost of various components for production of one ton of LIB [adapted and modified from a previous study (Gratz et al., 2014)]. LCO: LLiCoO_2_, NMC: LiNi_0.33_Mn_0.33_Co_0.33_O_2_, LMO: LiMn_2_O_4_, LNO: LiNiO_2_, and LFP: LiFePO_4_.


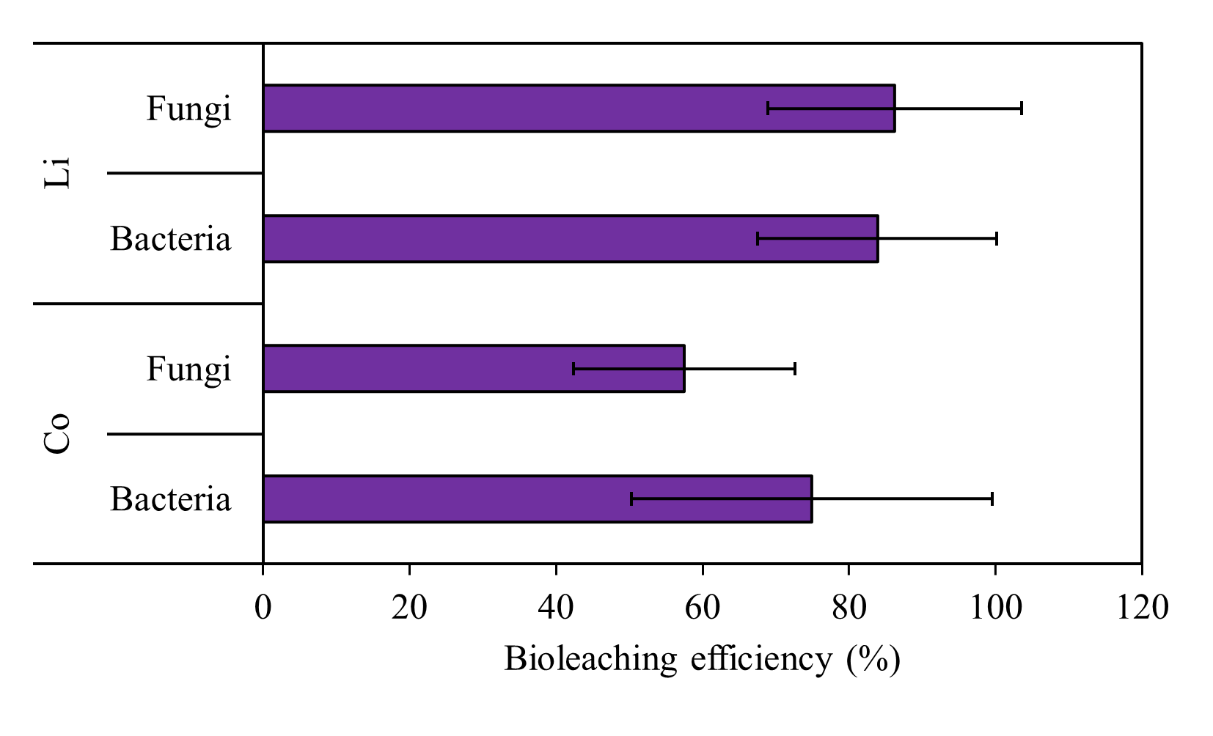


**Supplementary Figure 4.** Comparison of Co and Li dissolution efficiency between bacterial and fungal bioleaching systems using literature data. The literature data reported in Table 4 (bacterial leaching) and Table 5 (fungal leaching) in the main text are used for computation of the average and associated standard errors.


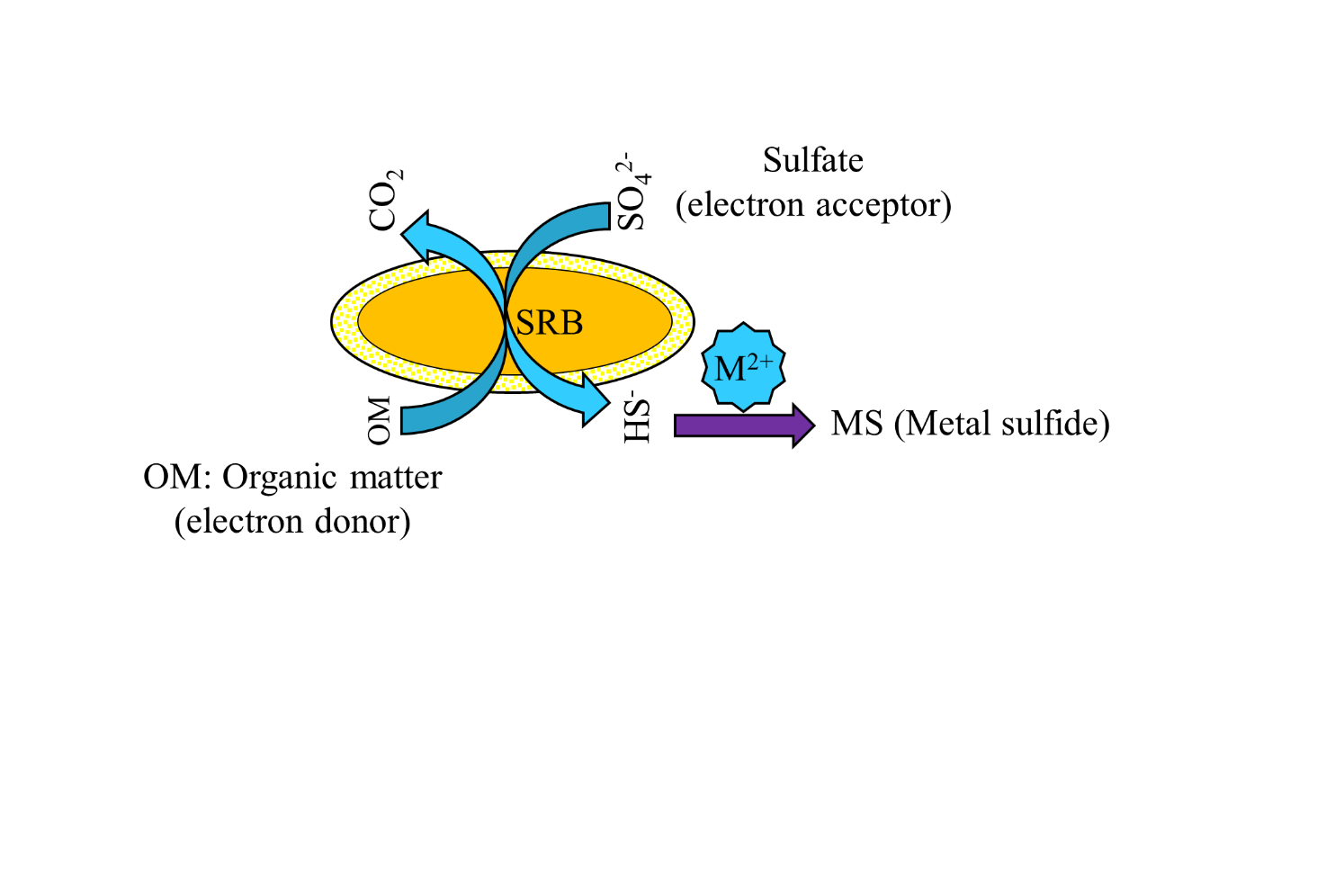


**Supplementary Figure 5.** Mechanism for sulfate reducing bacteria (SRB)-based bio-precipitation of metals.

## Supplementary Tables

| **Table S1.** Different components of a LIB and their weight fraction reported in various studies | | | | | | | |
| --- | --- | --- | --- | --- | --- | --- | --- |
|  | | Weight fraction (wt %) | | | | | |
| LIB components | (Georgi-Maschler et al., 2012) | | (Roy et al., 2022) | (Du et al., 2022) | (Alavi et al., 2021) | (Badawy et al., 2013) | www.soundguys .com |
| Case materials | 25 | | 26 | 30 | 34 | - | 30.2 |
| Cathode material | 25 | | 25 | 25 | 26 | 20 - 50 | 25.5 |
| Anode material | 17 | | 30 | 15 | 22 | 15 - 35 | 14.5 |
| Electrolyte | 10 | | 15 | 11 | - | 10 - 20 | 11.2 |
| Copper foil | 8 | | - | 8 | - | 3 - 12 | 8.1 |
| Aluminium foil | 5 | | - | 7 | - | 3 - 12 | 6.9 |
| Separator | 4 | | 4 | 4 | - | <8 | 3.6 |
| Plastics | - | | - | - | 15 | - | - |
| Others/Loss | 6 | | - | - | 3 | <5 | - |
| Total | 100 | | 100 | 100 | 100 | - | 100 |

| **Table S2.** Production of organic acids (bioacids) during bacterial and fungal bioleaching of spent LIBs | | | | | | | | | |  |
| --- | --- | --- | --- | --- | --- | --- | --- | --- | --- | --- |
| Microbial agent | Energy/Carbon source | Type of bioacid | Bioacid concentration | Additional information | | | | Reference | | |
| **Bacterial leaching** |  |  |  |  | | | |  | | |
| *A. ferrooxidans* (DSMZ, 1927) | FeSO_4,_ 150 g/L | H_2_SO_4_ | 0.52 M | One-step leaching | | | | (Roy et al., 2021) | | |
| *A, thiooxidans* (80191) | S^0^, 1% (w/v) | H_2_SO_4_ | 10.2 mM | Pure-culture medium | | | | (Biswal et al., 2018) | | |
| *A. thiooxidans* (80191) | S^0^, 1% (w/v) | H_2_SO_4_ | 1.7 mM | One-step leaching | | | | (Biswal et al., 2018) | | |
| *A. thiooxidans* (80191) | S^0^, 1% (w/v) | H_2_SO_4_ | 2.6 mM | Two-step leaching | | | | (Biswal et al., 2018) | | |
| **Fungal leaching** |  |  |  |  | | | |  | | |
| *A. niger* MM1 (Isolated) | 100 g/L sucrose | Citric acid | 102.4 mM | Pure-culture medium | | | | (Biswal et al., 2018) | | |
| *A. niger* MM1 (Isolated) | 100 g/L sucrose | Citric acid | 40.7 mM | One-step leaching | | | | (Biswal et al., 2018) | | |
| *A. niger* MM1 (Isolated) | 100 g/L sucrose | Citric acid | 70.8 mM | Two-step leaching | | | | (Biswal et al., 2018) | | |
| *A. niger* SG1 (Isolated) | 100 g/L sucrose | Citric acid | 76.9 mM | Pure-culture medium | | | | (Biswal et al., 2018) | | |
| *A. niger* SG1 (Isolated) | 100 g/L sucrose | Citric acid | 43.1 mM | One-step leaching | | | | (Biswal et al., 2018) | | |
| *A. niger* SG1 (Isolated) | 100 g/L sucrose | Citric acid | 59.5 mM | Two-step leaching | | | | (Biswal et al., 2018) | | |
| *A. niger* (Isolated) | 3 g/L Glucose | Citric acid | ~0.09 M | One-step leaching | | | | (Hariyadi et al., 2022) | | |
| *A. niger* (PTCC 5210) | 116.90 g/L sucrose | Citric acid | 26,478 mg/L | Spent medium leaching | | | | (Bahaloo-Horeh and Mousavi, 2017) | | |
| *A. niger* (PTCC 5210) | 116.90 g/L sucrose | Malic acid | 1832.53 mg/L | Spent medium leaching | | | | (Bahaloo-Horeh and Mousavi, 2017) | | |
| *A. niger* (PTCC 5210) | 116.90 g/L sucrose | Gluconic acid | 8433.76 mg/L | Spent medium leaching | | | | (Bahaloo-Horeh and Mousavi, 2017) | | |
| *A. niger* (PTCC 5210) | 116.90 g/L sucrose | Oxalic acid | 305.558 mg/L | Spent medium leaching | | | | (Bahaloo-Horeh and Mousavi, 2017) | | |
| *A. niger* (PTCC 5210) | 100 g/L sucrose | Oxalic acid | 9948 mg/L | One-step leaching | | | | (Horeh et al., 2016) | | |
| *A. niger* (PTCC 5210) | 100 g/L sucrose | Citric acid | 133 mg/L | Two-step leaching | | | | (Horeh et al., 2016) | | |
| *A. niger* (PTCC 5210) | 100 g/L sucrose | Oxalic acid | 3372 mg/L | Two-step leaching | | | | (Horeh et al., 2016) | | |
| *A. niger* (PTCC 5210) | 100 g/L sucrose | Gluconic acid | 102 mg/L | Two-step leaching | | | | (Horeh et al., 2016) | | |
| *A. niger* (PTCC 5210) | 100 g/L sucrose | Citric acid | 8078 mg/L | Spent medium leaching | | | | (Horeh et al., 2016) | | |
| *A. niger* (PTCC 5210) | 100 g/L sucrose | Oxalic acid | 1170 mg/L | Spent medium leaching | | | | (Horeh et al., 2016) | | |
| *A. niger* (PTCC 5210) | 100 g/L sucrose | Malic acid | 1251 mg/L | Spent medium leaching | | | | (Horeh et al., 2016) | | |
| *A. niger* (PTCC 5210) | 100 g/L sucrose | Gluconic acid | 2126 mg/L | Spent medium leaching | | | | (Horeh et al., 2016) | | |
| *A. niger* (PTCC 5010) | 20 g/L Glucose | Gluconic acid | 186.5 mM | Pure-culture medium | | | | (Kazemian et al., 2020) | | |
| *A. niger* (PTCC 5010) | 20 g/L Glucose | Oxalic acid | 11.6 mM | Pure-culture medium | | | | (Kazemian et al., 2020) | | |
| *A. niger* (PTCC 5010) | 20 g/L Glucose | Tartaric acid | 0 mM | Pure-culture medium | | | | (Kazemian et al., 2020) | | |
| *A. niger* (PTCC 5010) | 20 g/L Glucose | Malic acid | 0.427 mM | Pure-culture medium | | | | (Kazemian et al., 2020) | | |
| *A. niger* (PTCC 5010) | 20 g/L Glucose | Citric acid | 0.889 mM | | Pure-culture medium | | | | (Kazemian et al., 2020) | |
| *A. niger* (PTCC 5010) | 20 g/L Glucose | Gluconic acid | 328.5 mM | | One-step leaching | | | | (Kazemian et al., 2020) | |
| *A. niger* (PTCC 5010) | 20 g/L Glucose | Oxalic acid | 7.8 mM | | One-step leaching | | | | (Kazemian et al., 2020) | |
| *A. niger* (PTCC 5010) | 20 g/L Glucose | Tartaric acid | 0 mM | | One-step leaching | | | | (Kazemian et al., 2020) | |
| *A. niger* (PTCC 5010) | 20 g/L Glucose | Malic acid | 0.22 mM | | One-step leaching | | | | (Kazemian et al., 2020) | |
| *A. niger* (PTCC 5010) | 20 g/L Glucose | Citric acid | 0.857 mM | | One-step leaching | | | | (Kazemian et al., 2020) | |
| *P. chrysogenum* (PTCC 5037) | 20 g/L Glucose | Gluconic acid | 128.6 mM | | Pure-culture medium | | | | (Kazemian et al., 2020) | |
| *P. chrysogenum* (PTCC 5037) | 20 g/L Glucose | Oxalic acid | 34.59 mM | | Pure-culture medium | | | | (Kazemian et al., 2020) | |
| *P. chrysogenum* (PTCC 5037) | 20 g/L Glucose | Tartaric acid | 26.4mM | | Pure-culture medium | | | | (Kazemian et al., 2020) | |
| *P. chrysogenum* (PTCC 5037) | 20 g/L Glucose | Malic acid | 0 mM | | Pure-culture medium | | | | (Kazemian et al., 2020) | |
| *P. chrysogenum* (PTCC 5037) | 20 g/L Glucose | Citric acid | 4.6 mM | | Pure-culture medium | | | | (Kazemian et al., 2020) | |
| *P. chrysogenum* (PTCC 5037) | 20 g/L Glucose | Gluconic acid | 152.5 mM | | One-step leaching | | | | (Kazemian et al., 2020) | |
| *P. chrysogenum* (PTCC 5037) | 20 g/L Glucose | Oxalic acid | 62.28 mM | | One-step leaching | | | | (Kazemian et al., 2020) | |
| *P. chrysogenum* (PTCC 5037) | 20 g/L Glucose | Tartaric acid | 29.5 mM | | One-step leaching | | | | (Kazemian et al., 2020) | |
| *P. chrysogenum* (PTCC 5037) | 20 g/L Glucose | Malic acid | 0 mM | | One-step leaching | | | | (Kazemian et al., 2020) | |
| *P. chrysogenum* (PTCC 5037) | 20 g/L Glucose | Citric acid | 3.75 mM | | One-step leaching | | | | (Kazemian et al., 2020) | |
| *A. niger* (ATCC 6275) | 20 g/L dextrose | Oxalic acid | 2.3 mM | | Control (no metal) | | | | (Lobos et al., 2021) | |
| *A. niger* (ATCC 6275) | 20 g/L dextrose | Citric acid | 15.8 mM | | Control (no metal) | | | | (Lobos et al., 2021) | |
| *A. niger* (ATCC 6275) | 20 g/L dextrose | Tartaric acid | 14.8 mM | | Control (no metal) | | | | (Lobos et al., 2021) | |
| *A. niger* (ATCC 6275) | 20 g/L dextrose | L-malic acid | - | | Control (no metal) | | | | (Lobos et al., 2021) | |
| *A. niger* (ATCC 6275) | 20 g/L dextrose | Oxalic acid | 1.7 | | Addition of 50 mg/L Li | | | | (Lobos et al., 2021) | |
| *A. niger* (ATCC 6275) | 20 g/L dextrose | Citric acid | 8.7 | | Addition of 50 mg/L Li | | | | (Lobos et al., 2021) | |
| *A. niger* (ATCC 6275) | 20 g/L dextrose | Tartaric acid | - | | Addition of 50 mg/L Li | | | | (Lobos et al., 2021) | |
| *A. niger* (ATCC 6275) | 20 g/L dextrose | L-malic acid | - | | Addition of 50 mg/L Li | | | | (Lobos et al., 2021) | |
| *P. simplicissimum* (ATCC 48705) | 20 g/L dextrose | Oxalic acid | - | | Control (no metal) | | | | (Lobos et al., 2021) | |
| *P. simplicissimum* (ATCC 48705) | 20 g/L dextrose | Citric acid | 12.4 mM | | Control (no metal) | | | | (Lobos et al., 2021) | |
| *P. simplicissimum* (ATCC 48705) | 20 g/L dextrose | Tartaric acid | 8.3 mM | | Control (no metal) | | | | (Lobos et al., 2021) | |
| *P. simplicissimum* (ATCC 48705) | 20 g/L dextrose | L-malic acid | - | | Control (no metal) | | | | (Lobos et al., 2021) | |
| *P. simplicissimum* (ATCC 48705) | 20 g/L dextrose | Oxalic acid | 11.6 mM | | Addition of 50 mg/L Li | | | | (Lobos et al., 2021) | |
| *P. simplicissimum* (ATCC 48705) | 20 g/L dextrose | Citric acid | 4.1 mM | | Addition of 50 mg/L Li | | | | (Lobos et al., 2021) | |
| *P. simplicissimum* (ATCC 48705) | 20 g/L dextrose | Tartaric acid | - | | Addition of 50 mg/L Li | | | | (Lobos et al., 2021) | |
| *P. simplicissimum* (ATCC 48705) | 20 g/L dextrose | L-malic acid | - | | Addition of 50 mg/L Li | | | | (Lobos et al., 2021) | |
| *P. Chrysogenum* (ATCC 10108) | 20 g/L dextrose | Oxalic acid | - | | Control (no metal) | | | | (Lobos et al., 2021) | |
| *P. Chrysogenum* (ATCC 10108) | 20 g/L dextrose | Citric acid | - | | Control (no metal) | | | | (Lobos et al., 2021) | |
| *P. Chrysogenum* (ATCC 10108) | 20 g/L dextrose | Tartaric acid | - | | Control (no metal) | | | | (Lobos et al., 2021) | |
| *P. Chrysogenum* (ATCC 10108) | 20 g/L dextrose | L-malic acid | 19.0 mM | | Control (no metal) | | | | (Lobos et al., 2021) | |
| *P. Chrysogenum* (ATCC 10108) | 20 g/L dextrose | Oxalic acid | - | | Addition of 50 mg/L Li | | | | (Lobos et al., 2021) | |
| *P. Chrysogenum* (ATCC 10108) | 20 g/L dextrose | Citric acid | - | | Addition of 50 mg/L Li | | | | (Lobos et al., 2021) | |
| *P. Chrysogenum* (ATCC 10108) | 20 g/L dextrose | Tartaric acid | - | | Addition of 50 mg/L Li | | | | (Lobos et al., 2021) | |
| *P. Chrysogenum* (ATCC 10108) | 20 g/L dextrose | L-malic acid | 22.8 mM | | Addition of 50 mg/L Li | | | | (Lobos et al., 2021) | |
| Note: |  |  |  | |  | | | | |  |
| *A. ferrooxidans: Acidithiobacillus ferrooxidans* | |  |  | | |  | | | |  |
| *A. thiooxidans: Acidithiobacillus thiooxidans* | |  |  | | | |  | | |  |
| *A. niger: Aspergillus niger* |  |  |  | | | |  | | |  |
| *P. simplicissimum: Penicillium simplicissimum:* | |  |  | | | |  | | |  |
| *P. chrysogenum: Penicillium chrysogenum* | |  |  | | | |  | | |  |

| **Table S3.** Optimum leaching condition for recovery of valuable metals from spent LIBs | | |  |
| --- | --- | --- | --- |
| Parameters | Leaching microorganism | Metal dissolution efficiency | Reference |
| **pH** |  |  |  |
| 1.5 | *A. ferrooxidans* (Isolated) | Co: 47.6% | (Li et al., 2013) |
| 2.5 | *A. ferrooxidans* (ATCC 19859) | Co: ~56%; Li: ~10.5% | (Mishra et al., 2008) |
| 1.5 | Mixed culture 1 | Co: 50.4 %, Li: 99.2%, Ni: 89.4% | (Heydarian et al., 2018) |
| 1.5 | Mixed culture 1 | Co:67%, Li: 80% | (Marcincakova et al., 2016) |
| 5.44 | *A. niger* (PTCC 5210) | Co: 64%, Li: 100%, Cu: 100%, Mn: 77%, Al: 75%, Ni: 54% | (Bahaloo-Horeh and Mousavi, 2017) |
| **Substrate/energy source** | |  |  |
| Fe^2+^: 45 g/L | *A. ferrooxidans* (Isolated) | Co: 48.2% | (Li et al., 2013) |
| Fe^2+^: 3 g/L | *A. ferrooxidans* (ATCC 19859) | Co: ~56%; Li: ~10.5% | (Mishra et al., 2008) |
| FeSO_4_: 36.7 mg/L | Mixed culture 1 | Co: 50.4 %, Li: 99.2%, Ni: 89.4% | (Heydarian et al., 2018) |
| FeSO_4_: 24.25 mg/L | Mixed culture 2 | Co: 99.9%, Li: 84%, Ni: 99.7% | (Ghassa et al., 2020) |
| S^0^: 10 g/L | Mixed culture 2 | Co: 70%, Li: 90%, Ni: 97% | (Ghassa et al., 2020) |
| S^0^: 5 g/L | Mixed culture 1 | Co: 50.4 %, Li: 99.2%, Ni: 89.4% | (Heydarian et al., 2018) |
| S^0^: 1 g/L and Fe^2+^: 3 g/L | *A. ferrooxidans* (ATCC 19859) | C: 65%, Li: ~9.5% | (Mishra et al., 2008) |
|  |  |  |  |
| **Temperature** |  |  |  |
| 35 °C | Mixed culture 3 | Co: 72%, Li: 89% | (Niu et al., 2014) |
| 30 °C | *A. ferrooxidans* (ATCC 19859) | C: 65%, Li: ~9.5% | (Mishra et al., 2008) |
| **Pulp density** |  |  |  |
| 0.5 g/L | *A. ferrooxidans* (ATCC 19859) | Co: ~65%; Li: ~10.5% | (Mishra et al., 2008) |
| 30 g/L | *A. s thiooxidans* (PTCC 1717) | Co: 60%, Li: 99%, Mn: 20% | (Naseri et al., 2019b) |
| 40 g/L | *A. ferrooxidans* (PTCC 1647) | Co: 83%, Li: 100%, Mn: 20% | (Naseri et al., 2019a) |
| 2 g/L | Mixed culture 3 | Co: 72%, Li: 89% | (Niu et al., 2014) |
| 5% (w/v) | Mixed culture 4 | Co: 96.3%, Li: 98.1% | (Liu et al., 2020) |
| 2% (w/v) | *A. niger* (PTCC 5210) | Cu: 100%, Li: 100%, Mn: 77%, Al: 75% | (Bahaloo-Horeh and Mousavi, 2017) |
| 1% (w/v) | *A. niger* (PTCC 5210) | Co: 64%, Ni; 54% | (Bahaloo-Horeh and Mousavi, 2017) |
| **Catalyst** |  |  |  |
| Cu^2+^: 0.75 g/L | *A. ferrooxidans* (Isolated) | Co: 99.9% | (Zeng et al., 2012) |
| Ag+: 0.02 g/L | *A. ferrooxidans* (Isolated) | Co: 98.4% | (Zeng et al., 2013) |
| Ag+: 0.02 g/L | *A. ferrooxidans* (PTCC 1647) | Co: 99.95%, Ni: 99.95% | (Noruzi et al., 2022) |
| **Ultrasonic treatment** |  |  |  |
| 203.5 W for 30 minutes | *A. ferrooxidans*(PTCC 1647) | Co: 19%, Li: 67%, Mn: 50%, Ni: 34% | (Nazerian et al., 2023) |
| **Spent LIBs particle size** |  |  |  |
| 62 µm | *A. ferrooxidans* (PTCC 1647) | Co: 93.7%, Ni: 87%, Cd: 67% | (Ijadi Bajestani et al., 2014) |
| Note: |  |  |  |
| Mixed culture 1: *Acidithiobacillus ferrooxidans* and *Acidithiobacillus thiooxidans* | | |  |
| Mixed culture 2: *Acidithiobacillus caldus, Leptospirillum ferriphilum, Sulfobacillus* spp. and *Ferroplasma* spp. | | | |
| Mixed culture 3: *Alicyclobacillus* spp*.* and *Sulfobacillus* spp. | | | |
| Mixed culture 4: *Leptospirillum ferriphilum* and *Sulfobacillus thermosulfidooxidans* | | | |
| *A. ferrooxidans*: *Acidithiobacillus ferrooxidans* | |  |  |

**Reference**

Alavi, N., Partovi, K., Majlessi, M., Rashidi, M., and Alimohammadi, M. (2021). Bioleaching of metals from cellphones batteries by a co-fungus medium in presence of carbon materials. *Bioresour. Technol. Reports* 15, 100768. doi: https://doi.org/10.1016/j.biteb.2021.100768.

Badawy, S., Nayl, A., Elkhashab, R. A., and El-Khateeb, M. (2013). Cobalt separation from waste mobile phone batteries using selective precipitation and chelating resin. *J. Mater. Cycles Waste Manag.* 16. doi: 10.1007/s10163-013-0213-y.

Bahaloo-Horeh, N., and Mousavi, S. M. (2017). Enhanced recovery of valuable metals from spent lithium-ion batteries through optimization of organic acids produced by Aspergillus niger. *Waste Manag.* 60, 666–679. doi: https://doi.org/10.1016/j.wasman.2016.10.034.

Biswal, B. K., Jadhav, U. U., Madhaiyan, M., Ji, L., Yang, E.-H., and Cao, B. (2018). Biological leaching and chemical precipitation methods for recovery of Co and Li from spent lithium-ion batteries. *ACS Sustain. Chem. Eng.* 6, 12343–12352. doi: 10.1021/acssuschemeng.8b02810.

Du, K., Ang, E. H., Wu, X., and Liu, Y. (2022). Progresses in Sustainable Recycling Technology of Spent Lithium-Ion Batteries. *ENERGY \& Environ. Mater.* 5, 1012–1036. doi: https://doi.org/10.1002/eem2.12271.

Georgi-Maschler, T., Friedrich, B., Weyhe, R., Heegn, H., and Rutz, M. (2012). Development of a recycling process for Li-ion batteries. *J. Power Sources* 207, 173–182. doi: https://doi.org/10.1016/j.jpowsour.2012.01.152.

Ghassa, S., Farzanegan, A., Gharabaghi, M., and Abdollahi, H. (2020). Novel bioleaching of waste lithium ion batteries by mixed moderate thermophilic microorganisms, using iron scrap as energy source and reducing agent. *Hydrometallurgy* 197, 105465. doi: https://doi.org/10.1016/j.hydromet.2020.105465.

Gratz, E., Sa, Q., Apelian, D., and Wang, Y. (2014). A closed loop process for recycling spent lithium ion batteries. *J. Power Sources* 262, 255–262. doi: https://doi.org/10.1016/j.jpowsour.2014.03.126.

Hariyadi, A., Masago, A. R., Febrianur, R., and Rahmawati, D. (2022). Optimization Fungal Leaching of Cobalt and Lithium from Spent Li-Ion Batteries Using Waste Spices Candlenut. *Key Eng. Mater.* 938, 177–182. doi: 10.4028/p-lkr100.

Heydarian, A., Mousavi, S. M., Vakilchap, F., and Baniasadi, M. (2018). Application of a mixed culture of adapted acidophilic bacteria in two-step bioleaching of spent lithium-ion laptop batteries. *J. Power Sources* 378, 19–30. doi: https://doi.org/10.1016/j.jpowsour.2017.12.009.

Horeh, N. B., Mousavi, S. M., and Shojaosadati, S. A. (2016). Bioleaching of valuable metals from spent lithium-ion mobile phone batteries using Aspergillus niger. *J. Power Sources* 320, 257–266. doi: https://doi.org/10.1016/j.jpowsour.2016.04.104.

Ijadi Bajestani, M., Mousavi, S. M., and Shojaosadati, S. A. (2014). Bioleaching of heavy metals from spent household batteries using Acidithiobacillus ferrooxidans: Statistical evaluation and optimization. *Sep. Purif. Technol.* 132, 309–316. doi: https://doi.org/10.1016/j.seppur.2014.05.023.

Kazemian, Z., Larypoor, M., and Marandi, R. (2020). Evaluation of myco-leaching potential of valuable metals from spent lithium battery by Penicillium chrysogenum and Aspergillus niger. *Int. J. Environ. Anal. Chem.* 103, 514–527. doi: 10.1080/03067319.2020.1861605.

Li, L., Zeng, G., Luo, S., Deng, X., and Xie, Q. (2013). Influences of solution pH and redox potential on the bioleaching of LiCoO2 from spent lithium-ion batteries. *J. Korean Soc. Appl. Biol. Chem.* 56, 187–192. doi: 10.1007/s13765-013-3016-x.

Liu, X., Liu, H., Wu, W., Zhang, X., Gu, T., Zhu, M., et al. (2020). Oxidative Stress Induced by Metal Ions in Bioleaching of LiCoO2 by an Acidophilic Microbial Consortium. *Front. Microbiol.* 10. doi: 10.3389/fmicb.2019.03058.

Lobos, A., Harwood, V. J., Scott, K. M., and Cunningham, J. A. (2021). Tolerance of three fungal species to lithium and cobalt: Implications for bioleaching of spent rechargeable Li-ion batteries. *J. Appl. Microbiol.* 131, 743–755. doi: https://doi.org/10.1111/jam.14947.

Marcincakova, R., Kadukova, J., Mrazikova, A., Velgosova, O., Luptakova, A., and Ubaldini, S. (2016). Metal Bioleaching from Spent Lithium-Ion Batteries Using Acidophilic Bacterial Strains. *Inżynieria Miner.* R. 17, nr, 117–120.

Mishra, D., Kim, D.-J., Ralph, D. E., Ahn, J.-G., and Rhee, Y.-H. (2008). Bioleaching of metals from spent lithium ion secondary batteries using Acidithiobacillus ferrooxidans. *Waste Manag.* 28, 333–338. doi: https://doi.org/10.1016/j.wasman.2007.01.010.

Naseri, T., Bahaloo-Horeh, N., and Mousavi, S. M. (2019a). Bacterial leaching as a green approach for typical metals recovery from end-of-life coin cells batteries. *J. Clean. Prod.* 220, 483–492. doi: https://doi.org/10.1016/j.jclepro.2019.02.177.

Naseri, T., Bahaloo-Horeh, N., and Mousavi, S. M. (2019b). Environmentally friendly recovery of valuable metals from spent coin cells through two-step bioleaching using Acidithiobacillus thiooxidans. *J. Environ. Manage.* 235, 357–367. doi: https://doi.org/10.1016/j.jenvman.2019.01.086.

Nazerian, M., Bahaloo-Horeh, N., and Mousavi, S. M. (2023). Enhanced bioleaching of valuable metals from spent lithium-ion batteries using ultrasonic treatment. *Korean J. Chem. Eng.* 40, 584–593. doi: 10.1007/s11814-022-1257-2.

Niu, Z., Zou, Y., Xin, B., Chen, S., Liu, C., and Li, Y. (2014). Process controls for improving bioleaching performance of both Li and Co from spent lithium ion batteries at high pulp density and its thermodynamics and kinetics exploration. *Chemosphere* 109, 92–98. doi: https://doi.org/10.1016/j.chemosphere.2014.02.059.

Noruzi, F., Nasirpour, N., Vakilchap, F., and Mousavi, S. M. (2022). Complete bioleaching of Co and Ni from spent batteries by a novel silver ion catalyzed process. *Appl. Microbiol. Biotechnol.* 106, 5301–5316. doi: 10.1007/s00253-022-12056-0.

Roy, J. J., Madhavi, S., and Cao, B. (2021). Metal extraction from spent lithium-ion batteries (LIBs) at high pulp density by environmentally friendly bioleaching process. *J. Clean. Prod.* 280, 124242. doi: https://doi.org/10.1016/j.jclepro.2020.124242.

Roy, J. J., Rarotra, S., Krikstolaityte, V., Zhuoran, K. W., Cindy, Y. D.-I., Tan, X. Y., et al. (2022). Green Recycling Methods to Treat Lithium-Ion Batteries E-Waste: A Circular Approach to Sustainability. *Adv. Mater.* 34, 2103346. doi: https://doi.org/10.1002/adma.202103346.

Zeng, G., Deng, X., Luo, S., Luo, X., and Zou, J. (2012). A copper-catalyzed bioleaching process for enhancement of cobalt dissolution from spent lithium-ion batteries. *J. Hazard. Mater.* 199–200, 164–169. doi: https://doi.org/10.1016/j.jhazmat.2011.10.063.

Zeng, G., Luo, S., Deng, X., Li, L., and Au, C. (2013). Influence of silver ions on bioleaching of cobalt from spent lithium batteries. *Miner. Eng.* 49, 40–44. doi: https://doi.org/10.1016/j.mineng.2013.04.021.
